# Improved state‑transition analysis in tobacco lines differing in isoprene emission via spectral‑retrieval separation of PSI and PSII fluorescence at physiological temperature

**DOI:** 10.1007/s11120-026-01227-z

**Published:** 2026-07-23

**Authors:** Lorenzo Palombi, Susanna Pollastri, Francesco Loreto, Giovanni Agati

**Affiliations:** 1 Institute of Applied Physics “Nello Carrara” (IFAC), National Research Council (CNR), 50019 Sesto Fiorentino, Italy; 2 Institute for Sustainable Plant Protection (IPSP), National Research Council (CNR), 50019 Sesto Fiorentino, Italy; 3 Department of Biology, University of Naples Federico II, 80126 Naples, Italy

**Keywords:** Chlorophyll, Fluorescence emission spectra, *Nicotiana tabacum*, Photosystem I, Photosystem II, State transition rate, State transition extent

## Abstract

State transition (ST) is a key regulatory mechanism that balances excitation energy between photosystem II (PSII) and photosystem I (PSI). Although its general principles have been known for decades, methodological constraints have limited precise quantification of ST kinetics and of the associated changes in photosystem absorption cross sections. We present a PCA‑assisted fluorescence spectral analysis that enables in vivo separation of PSI and PSII chlorophyll fluorescence at physiological temperatures. Applied to intact *Nicotiana tabacum* leaves undergoing ST, this method provides more accurate estimates of both the rates and the extent of the process than earlier approaches. The half‑time of the State 1 to State 2 (St1→St2) transition was approximately 1 min, nearly twice as fast as values inferred from traditional F_far‑red_/F_red_ fluorescence ratios measured at room temperature or 77 K. The analysis also clarifies how PSI and PSII fluorescence changes relate to variations in their absorption cross sections. Accounting for PSII‑to‑PSI spillover, we found that the mobile LHCII fraction associating with PSI during the St1→St2 transition increased the PSI absorption cross section by 31%, whereas LHCII reassociation with PSII during the reverse transition produced a more modest 17% increase in PSII absorption cross section. These asymmetric adjustments likely have important physiological implications under fluctuating environmental conditions. Finally, applying the method to isoprene‑emitting and non‑emitting tobacco lines revealed that isoprene accelerates excitation‑energy redistribution during the St2→St1 transition, consistent with its proposed role in maintaining optimal thylakoid ultrastructure and fluidity. Overall, this approach offers a powerful tool for dissecting photosynthetic energy‑distribution dynamics in vivo.

**Supplementary information:**

The online version contains supplementary material available at 10.1007/s11120-026-01227-z.

## Introduction

In photoautotrophic organisms, state transition (ST) represents a key short‑term regulatory mechanism that balances the excitation energy between photosystem II (PSII) and photosystem I (PSI), thereby optimizing linear electron flow driving photosynthesis and photorespiration. Since its discovery more than 55 years ago (Bonaventura and Myers 1969; Murata 1969), ST has been extensively investigated. The biochemical mechanisms controlling ST seem to reside in the phosphorylation and dephosphorylation of specific light-harvesting complex II (LHCII) components. The ST process is regulated by the opposing activities of the State TransitioN 7 (STN7) kinase (Bellafiore et al. 2005) and the Protein PHosphatase 1/Thylakoid-Associated Phosphatase of 38 kDa (PPH1/TAP38) phosphatase (Pribil et al. 2010; Shapiguzov et al. 2010). These enzymes induce a reorganization of the thylakoid membrane, including changes in grana diameter and stacking (Wood and Johnson 2020), the formation of PSII–PSI megacomplexes that facilitate PSII‑to‑PSI spillover (Yokono et al. 2019), and the migration of a fraction of LHCII between the two photosystems. However, the relative importance of changes in the photosystem absorption cross‑sections and PSII‑to‑PSI spillover is still debated (Ruban and Johnson 2009; Terashima et al. 2025).

It is known that ST can be modulated using different light wavebands (Ruban and Johnson 2009). PSII preferentially absorbed light (blue-red light), hereafter referred to as ‘PSII light’, induces State 1 to State 2 (St1→St2) transition, which can be reversed (St2→St1) adding a far-red (FR) light (λ >700 nm), preferentially absorbed by PSI, hereafter referred as ‘PSI light’. PSII and PSI chlorophyll fluorescence (ChlF) components vary inversely, increasing or decreasing depending on the direction of ST changes (McCormac et al. 1994). This is due first to the reciprocal modulation of the two photosystem absorption capacity (Kyle et al. 1984), but also to changes in the PSII photochemical quenching (qP) resulting from shifts in the efficiency of the electron flow between the two photosystems (Taylor et al. 2019).

ST occurrence can therefore be tracked via ChlF measurements during shifts between blue–red and far-red radiation. This is commonly achieved using pulse-amplitude-modulated (PAM) fluorimetry to measure the ratio of maximum fluorescence in State 1 and State 2 (F_m1_/F_m2_), which serves as an index of ST (Haldrup et al. 2001). However, the F_m1_/F_m2_ index underestimates ST because the PSI contribution to Fm is non-negligible (Pfündel 1998; Agati et al. 2000), and cannot be discerned from the PSII fluorescence.

Fluorescence spectroscopy at 77 K has also been widely employed to assess ST, as this technique is able to separate the PSII red and PSI far‑red emission bands (McCormac et al. 1994; Tan et al. 1998; Ruban and Johnson 2009; Pribil et al. 2010; Vetoshkina et al. 2024). Nevertheless, recent work has demonstrated that even at liquid‑nitrogen temperature a vibronic contribution of PSII persists in the far‑red ChlF region, and this component must be considered to accurately deconvolute the two photosystem fluorescence signals (Terashima et al. 2025).

Time‑resolved fluorescence spectroscopy on intact leaves is a second powerful approach for separating the contributions of PSII and PSI to ChlF and properly quantify ST (Bos et al. 2019). Fluorescence decay components on the nanosecond timescale originate from PSII, whereas PSI‑associated fluorescence decays within < 100 ps. This assignment is supported by decay‑associated spectra obtained through multi‑wavelength time‑resolved fluorescence detection in the red and far‑red regions (Chukhutsina et al. 2019).

An alternative approach to retrieve the PSII and PSI spectral components of leaf ChlF at physiological temperatures was proposed early by Palombi et al. (2011). The method relies on Principal Component Analysis (PCA) of full ChlF emission spectra recorded under conditions in which the PSI spectral component remains constant while the PSII component varies in intensity. Under these conditions, the first principal component (PC1) provides a robust approximation of the PSII fluorescence emission spectrum, whereas the PSI spectrum can be estimated by subtracting the PC1 spectrum from the measured ChlF spectra. Reliable fitting of leaf ChlF spectra as a linear combination of the retrieved PSII and PSI spectral shapes then enables investigation of the dynamics of energy distribution between the two photosystems. PCA fluorescence spectroscopy allows investigation of ST in intact leaves at physiological temperatures, through a simpler, less equipment‑intensive, and faster approach than methods based on 77 K steady‑state or time‑resolved fluorescence. Moreover, large sets of ChlF spectral and temporal data can be acquired by PCA fluorescence spectroscopy, likely permitting an additional characterization of the dynamics of the ST process.

The rate of ST also depends on the viscosity of the thylakoid membranes (Vetoshkina et al. 2024). Isoprene is a volatile organic compound, synthetized in the plant chloroplasts and enabling protection of the photosynthetic apparatus from abiotic stresses (Pollastri et al. 2021). Due to its lipophilic nature, isoprene can intercalate inside the thylakoid membranes, maintaining their elasticity and fluidity. This effect was observed at physiological temperatures and was enhanced when plants experienced high temperatures (Pollastri et al. 2019). Thus, isoprene can facilitate molecular diffusion, electron transport rate and molecular reorganization of the thylakoid membranes, also reducing energy dissipation by the non-photochemical quenching (NPQ) of ChlF at physiological temperatures and under heat stress episodes (Pollastri et al. 2014). Because of all these structural changes induced by isoprene, and considering that ST can also be triggered by moderate heat stress (Nellaepalli et al. 2011), a role of isoprene in modulating ST mechanisms can be hypothesized.

Here, we applied the PCA fluorescence spectroscopy method to two tobacco (*Nicotiana tabacum* cv. Samsun) transgenic lines, emitting or non-emitting isoprene. The objectives of our study were: (i) to assess the usefulness of PCA fluorescence spectroscopy for characterizing ST changes, in comparison with previously established techniques; and (ii) to determine whether physiological changes associated with the presence of isoprene could affect ST.

## Material and methods

### Plant material

An isoprene-emitting transgenic tobacco (*N. tabacum*, cv. Samsun) homozygous (H) line, and the corresponding isoprene non-emitting azygous (A) line, which served as the ‘wild-type’ control equivalent, were used for this study. The lines were prepared inserting in the genome a vector carrying the isoprene synthase gene catalysing isoprene emission (H), or the empty vector only (A), as shown by Vickers et al. (2009). Ten days after seed germination, the seedlings were transferred to 2 L pots filled with a Klasmann compost (Klasmann-Deilmann GmbH, Geeste, Germany). Plants were cultivated in a growth chamber with air temperature between 20 and 25 °C and 40–60% relative humidity. The day/night photoperiod was set at 14 h/10 h, with a day light intensity of 500 µmol m^−2^s^−1^ photosynthetic photon flux density (PPFD). Plants were irrigated daily to field capacity of the pots. Six-week-old plants were then transferred outdoors under about 400 µmol m^−2^s^−1^ of diffuse solar radiation in order to expose them to natural temperature fluctuations. Within a 2-week period the plants experienced minimal, maximal and average air temperatures of 20.7 ± 1.4, 34.7 ± 2.1 and 27.3 ± 1.3, respectively. Fully expanded and intact leaves of eight-week-old plants were sampled on all non-destructive experiments.

### Chlorophyll fluorescence imaging

Pulse-Amplitude Modulation (PAM) images of chlorophyll fluorescence were obtained by a MAXI-Imaging-PAM Chlorophyll Fluorometer (Walz, Effeltrich, Germany). Leaf samples were illuminated at a distance of 20 cm by an array of blue (450 nm) light emitting diodes (LEDs) providing the pulse-modulated measuring light and the actinic illumination and saturation pulses. According to the manufacturer’s manual, at the standard working distance, the maximal deviation from the mean intensity is ±7%. This value was considered as the expected upper bound of illumination spatial inhomogeneity. The charge-coupled device (CCD) camera (Stingray F033B ASG, Allied Vision Technologies GmbH, Stadtroda, Germany) had a resolution of 640 × 480 pixels and was protected by a 3 mm RG645 Schott color glass filter plus a 770 nm short-pass filter. The camera integrated the ChlF signal at wavelengths > 645 nm and <770 nm.

Two tobacco leaves attached to the plant, one from H and the other from A line, were imaged simultaneously. For each leaf, the induction of the St2 to St1 transition was seen comparing spots (circular white area-of-interest (AOI) in Fig. S1) additionally irradiated by a FR light emitting diode (LED) at 720 nm (ELD-720–524, Roithner LaserTechnik GmbH, Vienna Austria) with those illuminated by the light source exciting preferentially PSII (black AOI in Fig. S1).

The following sequence of measurements was used. Plants were dark-adapted for at least 20 min and then the minimal, F_0_, and maximal F_m_ (under a 5000 µmol m^−2^ s^−1^ saturating pulse of light) Chl fluorescence were measured. Next, the PSII blue actinic light (35 µmol m^−2^ s^−1^) was switched on to induce the typical ChlF transient (known as the Kautsky effect) characterized by a rapid rise in fluorescence from PSII, followed by a slow decline. This first ChlF kinetics was measured for about 15 min. Next, the actinic light was switched off, to measure $F_0^\prime$ (along with a short pulse of FR light), and on again just before a saturating pulse was applied to measure the maximum fluorescence in illuminated leaves at St2, $F_{m2}^\prime$. Thereafter, the FR light was added for about further 15 min in the circular white AOI (Fig. S1), until St1 was reached. At this stage, the routine for measuring $F_0^\prime$ and $F_m^\prime$ was again executed, but this time the maximum fluorescence at St1 ($F_{m1}^\prime$), was measured. PSI light was then switched off for further 15 min to reach a new St2 when $F_0^\prime$ and $F_{m2}^\prime$ were again recorded.

The intensity of the FR LEDs was around 70 µmol m^−2^ s^−1^, measured by an OPHIR PD300-UV Silicon Photodiode Sensor (Ophir Optronics Solutions Ltd., Villanova di Castenaso (BO), Italy).

To assess the spatial homogeneity of the far-red illumination across the illuminated AOIs, we performed a simulation incorporating the main optical and geometrical characteristics of the LED source, including the specific properties of the LED used, a Lambertian angular emission pattern, and the inclination of the source relative to the sample plane. The simulation predicted a coefficient of variation (CV) of intensity between 5 and 9% across the illuminated AOIs.

Reference AOIs were selected outside the effective far-red illumination field. Direct PPFD measurements performed in these regions showed that the far-red contribution was negligible. This was further supported by the simulation, which indicated that far-red radiation in the reference AOIs corresponded to only 0.01–0.2% of that in the illuminated area.

The Imaging-PAM measurements were used to calculate the maximum quantum yield of photosystem II (PSII) photochemistry in darkened leaves $$({F_v}$$/$${F_m} = 1 - {F_0}/{F_m})$$, the photochemical efficiency of PSII in illuminated leaves (Φ_PSII_ = 1 − $${F_s}$$/$$F_m^\prime$$) (Genty et al. 1989) and the nonphotochemical quenching NPQ = $${F_m}$$/$$F_m^\prime$$-1 (Bilger and Björkman 1991). The fraction of open PSII reaction centres,qL (the primary electron acceptor Q_A_ in its oxidized state), was calculated according to Kramer et al. (2004), as qL = ($$F_m^\prime - {F_s}$$)/( $$F_m^\prime - F_0^\prime) \cdot F_0^\prime/{F_s}$$.

**Fig. 1:**
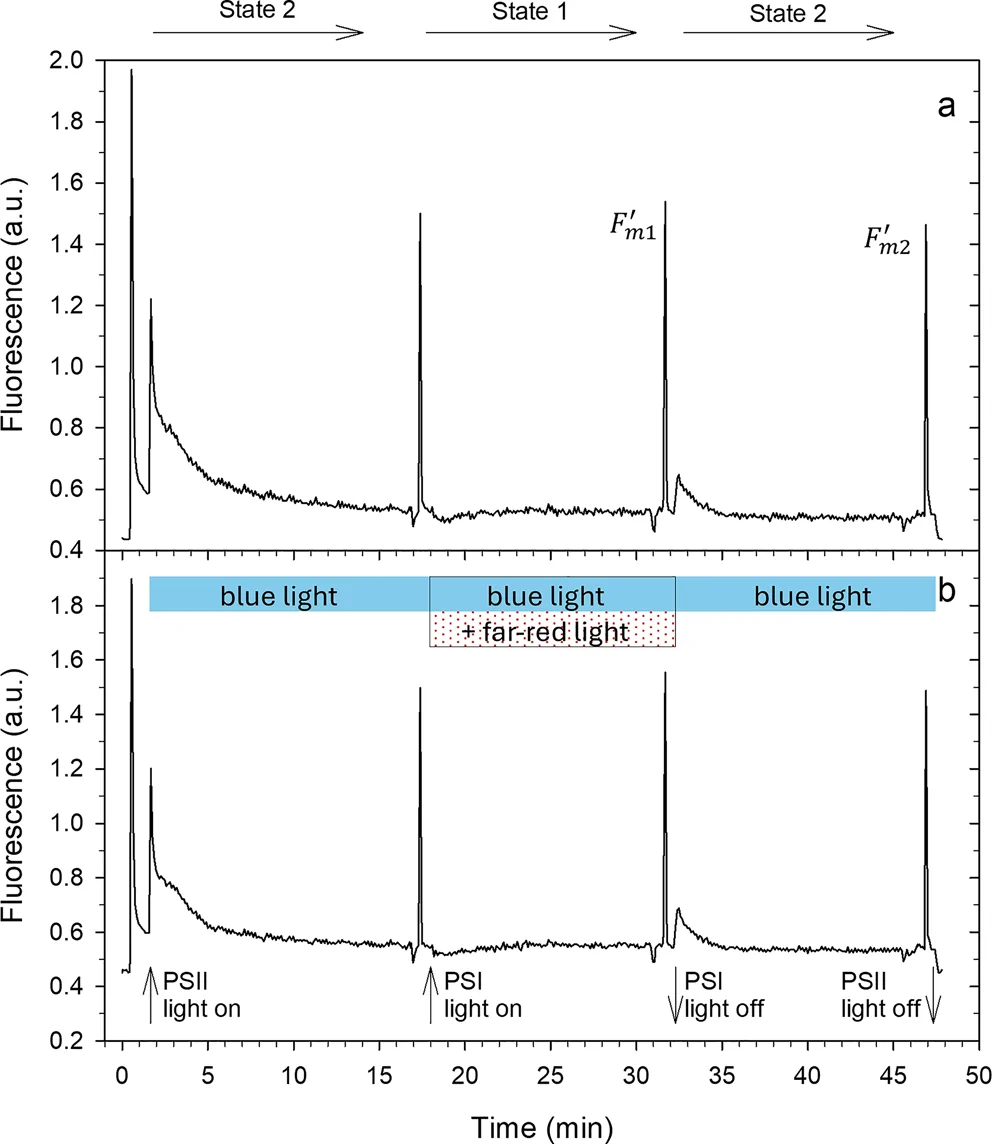
Representative kinetics of ChlF measured by the Imaging-PAM during state transition in an azygous A line (**a**) and isoprene-emitting H line (**b**) tobacco leaves

The extent of state transition was evaluated, as previously reported (Ruban and Johnson 2009), by the change in the maximal fluorescence between St1 and St2 relative to the value at St1, qT = [($F_{m1}^\prime$ – $F_{m2}^\prime$)/$F_{m1}^\prime$] x 100. The fluorescence signals used to determine all State 2 parameters were those recorded at the end of the final St1 to St2 transition (at approximately 45 min; Fig. 1).

### Chlorophyll fluorescence spectroscopy

Fluorescence spectra were acquired on the adaxial side of *N. tabacum* leaves attached to the plant, as reported in Fig. S2. A blue emitting LED (Nichia NSPB510AS, 465 nm, FWHM 20 nm @20 mA) at a PPFD of 30 µmol m^-2^ s^-1^ was used as both the probe exciting ChlF and the PSII actinic light to induce St2. An additional irradiation was provided by the 720 nm FR LED described above to preferentially excite PSI, when the shift to St1 was required. The intensity of both LEDs was measured by an OPHIR PD300-UV Silicon Photodiode Sensor (Ophir Optronics Solutions Ltd., Villanova di Castenaso (BO), Italy).

The fluorescence signal was collected by an optical fibre bundle (50 fibres, each with a diameter of 100 µm) at a distance of 1 cm from the leaf and at 90° with respect to the leaf surface, in a 45° geometry with respect to the excitation light.

After filtering through a long pass RG630 Schott filter in order to remove residual excitation light, the fluorescence was detected by means of an ARC SpectraPro 2300i spectrometer (300 mm of focal length and equipped with a 150-grooves/mm grating) coupled to a PI-MAX (Princeton Instrument) -intensified, gateable CCD (Thomson 7895, 512 × 512 pixels, 24 × 24 µm effective pitch). The system provided a 250‑nm spectral window between 600 and 850 nm with a nominal resolution of 0.5 nm.

Before measurements, leaves were dark‑adapted for 30 min. A sequence of fast fluorescence rise kinetics was then recorded by switching on only the blue LED. Each ChlF spectrum was acquired with an integration time of 10 ms. At first, 30 consecutive fast kinetics were collected at a frequency of 50 Hz; each was separated by a 20 s dark‑readaptation interval, to re-oxidize the primary electron acceptor Q_A_- (Chylla et al. 1987; Krause and Weis 1991), during which the blue LED was switched off.

After the last fast kinetic, and following an additional 20 s dark‑readaptation, a full St2‑inducing kinetic was recorded under continuous blue illumination. This kinetic lasted 20 min and spectra were acquired every 200 ms (5 Hz).

At the end of these 20 min, when the leaf had reached St2, the FR LED (720 nm) was added to promote the transition to St1. Because the FR emission overlapped with the detection spectral range, fluorescence spectra were recorded every 1000 ms with an exposure time of 10 ms, and the FR LED was switched off only during the 10‑ms detector exposures. In this configuration, the sample received FR illumination 99% of the time, while avoiding contamination by reflected FR signals on the detected fluorescence. This St1‑inducing phase also lasted 20 min.

Two additional 20‑min kinetics were subsequently measured: the first with only the blue LED switched on (blue‑only phase), and the second with both blue and FR LEDs illuminated (combined phase), using the same acquisition settings described for the full kinetics.

Kinetics of the ChlF and the F_740_/F_685_ ChlF ratio were derived by integrating the signals over the whole spectrum or 10-nm wide bands centered at 740 nm and 685 nm, respectively. Both signals were normalized to the values at the end of the first St2 (after 20 min from time zero).

### Chlorophyll fluorescence spectral analysis

The fluorescence spectral analysis method was described in details previously (Palombi et al. 2011).

Briefly, the leaf ChlF can be expressed as function of time and wavelength under conditions in which the intensity of the PSI component remains constant, as:

1 $$ChlF\left( {t,\lambda } \right) = {h_c} \cdot PSI\left( \lambda \right) + g\left( t \right) \cdot PSII\left( \lambda \right)$$

where PSI(λ) and PSII(λ) represent the fluorescence spectral shapes of the two photosystems, and ${h_c}$ and $g\left( t \right)$ are the intensities of PSI and PSII fluorescence, respectively. It should be noted that the PSI(λ) and PSII(λ) spectral shapes inherently include the effect of partial reabsorption of fluorescence by chlorophyll, which modifies the emission profiles in the red region.

Collecting the ChlF spectra during a time transient and applying the PCA at this data set, the whole information content from all the available spectral channels was exploited. With the assumption that a single spectral shape exists, whose intensity varies over time, the PSII(λ) spectrum could be determined from the first principal component (PC1). The PSI(λ) spectrum was derived by difference from the whole leaf ChlF spectrum with the assumption that its contribution in the range 605–675 nm is negligible (Palombi et al. 2011). The presence of more than one statistically significant principal component would indicate that the selected time interval of spectral acquisition does not satisfy the assumptions required for the applicability of the retrieval method, as multiple independent sources of variance would be present. In PCA, several procedures exist to assess component significance—such as Bartlett’s test for sphericity (Bartlett 1951), Horn’s parallel analysis (Horn 1965), and Broken‑stick criteria (Jackson 1993)—but all of them evaluate significance under the assumption that the dataset consists of independent and identically distributed observations and do not account for temporal autocorrelation in the data matrix (Zhang and Tong 2022).

Because standard PCA significance tests ignore time ordering, a component may appear statistically non‑significant in the global variance structure while still exhibiting a systematic, time‑correlated pattern in its scores. Such behavior indicates that the component, although small in total explained variance, captures a residual structured signal rather than pure noise. For this reason, relying exclusively on classical significance tests may lead to discarding components that retain physiologically meaningful temporal information.

To overcome this limitation, we complemented the statistical test (Bartlett’s procedure) with direct inspection of the temporal evolution of the second principal component (PC2) scores. Even if PC2 accounts for only a very small fraction of the total variance, the presence of a coherent, non‑random temporal structure in its scores must be considered, as this may indicate a slight violation of the model assumptions underlying the retrieval of the PSII component, and could therefore affect the accuracy of its determination. As an additional objective check for residual temporal structure, we applied the Ljung–Box portmanteau test to the PC2 score time series, which evaluates the null hypothesis of no serial autocorrelation up to a specified lag order (Ljung and Box 1978).

Given the full temporal range of fluorescence kinetics, our goal was therefore to identify the longest possible acquisition sub‑interval, extending from time t_start_ to t_end_, in which only one principal component is statistically significant and the second component shows no residual temporal autocorrelation. Maximizing the interval length is crucial because (i) it increases the number of spectra used to estimate the PSII spectral shape (PC1), improving the signal-to-noise ratio, and (ii) it allows us to account for as much variance as possible under the assumption that a single varying spectral component (PSII) dominates the kinetics.

For this reason, the optimal interval was selected through a two‑step criterion:

(i) verifying dimensionality reduction using Bartlett’s test (Bartlett 1951), and
(ii) confirming the absence of structured temporal patterns in the PC2 scores using the Ljung–Box portmanteau test.

To assess the robustness of the PCA-based retrieval method with respect to the choice of the analysis temporal window, a sensitivity analysis was performed by varying independently the start and end times of the selected temporal interval. For each resulting time window, the retrieval procedure was repeated, and the corresponding diagnostic parameters were recalculated, namely qT_PSI_, qT_PSII_, and the ratio between the 740 nm and 685 nm bands of the retrieved PSII spectrum (as index of its shape). The variability associated with the interval selection was then quantified, for each parameter X, as its coefficient of variation (CV), expressed as CV(X)=SD(X)/Mean(X) across the tested time windows.

### PSII and PSI contributions to Chl fluorescence during state transition

Once the two photosystem fluorescence spectral shapes were determined, any leaf ChlF spectrum could be considered as their weighted sum,

2 $$ChlF\left( \lambda \right) = {k_I} \cdot PSI\left( \lambda \right) + {k_{II}} \cdot PSII\left( \lambda \right)$$

and then the values of the ${k_I}$ and ${k_{II}}$ coefficients, reflecting the two photosystem contributions, can be retrieved by applying a least squares fit (LSF), using Eq. 2, to the measured spectra. This fitting was applied to all ChlF spectra recorded during the different ST time intervals. The derived PSI and PSII ChlF components were smoothed and their time evolution fitted by sigmoidal or exponential functions. The goodness of the fitting was evaluated by the coefficient of determination, r^2^. From the fitting parameters, the rate of change of the PSI and PSII fluorescence components during St2→St1 and vice versa, expressed as half-time, t_1/2_, of the process, and the estimated time, t_∞_, to reach their fluorescence steady state level at St2 and St1 were obtained. The extent of state transition was determined as the change of the PSII and PSI fluorescence components between St2 and St1 relative to the values at St2, that is qT_PSII_=(F_PSII-St1_– F_PSII-St2_)/F_PSII-St2_ and qT_PSI_=(F_PSI-St1_– F_PSI-St2_)/F_PSI-St2_.

### Statistical analysis

The statistical analysis related to the determination of the PSI and PSII fluorescence components were performed using MATLAB R2024a (MathWorks Inc., USA). Curve fitting procedures and graphical representations were carried out using SigmaPlot for Windows, Version 14.0 (Systat Software Inc., San Jose, CA, USA). Mean values were compared by t-test, and differences were considered statistically significant at *p* ≤ 0.05.

**Fig. 2:**
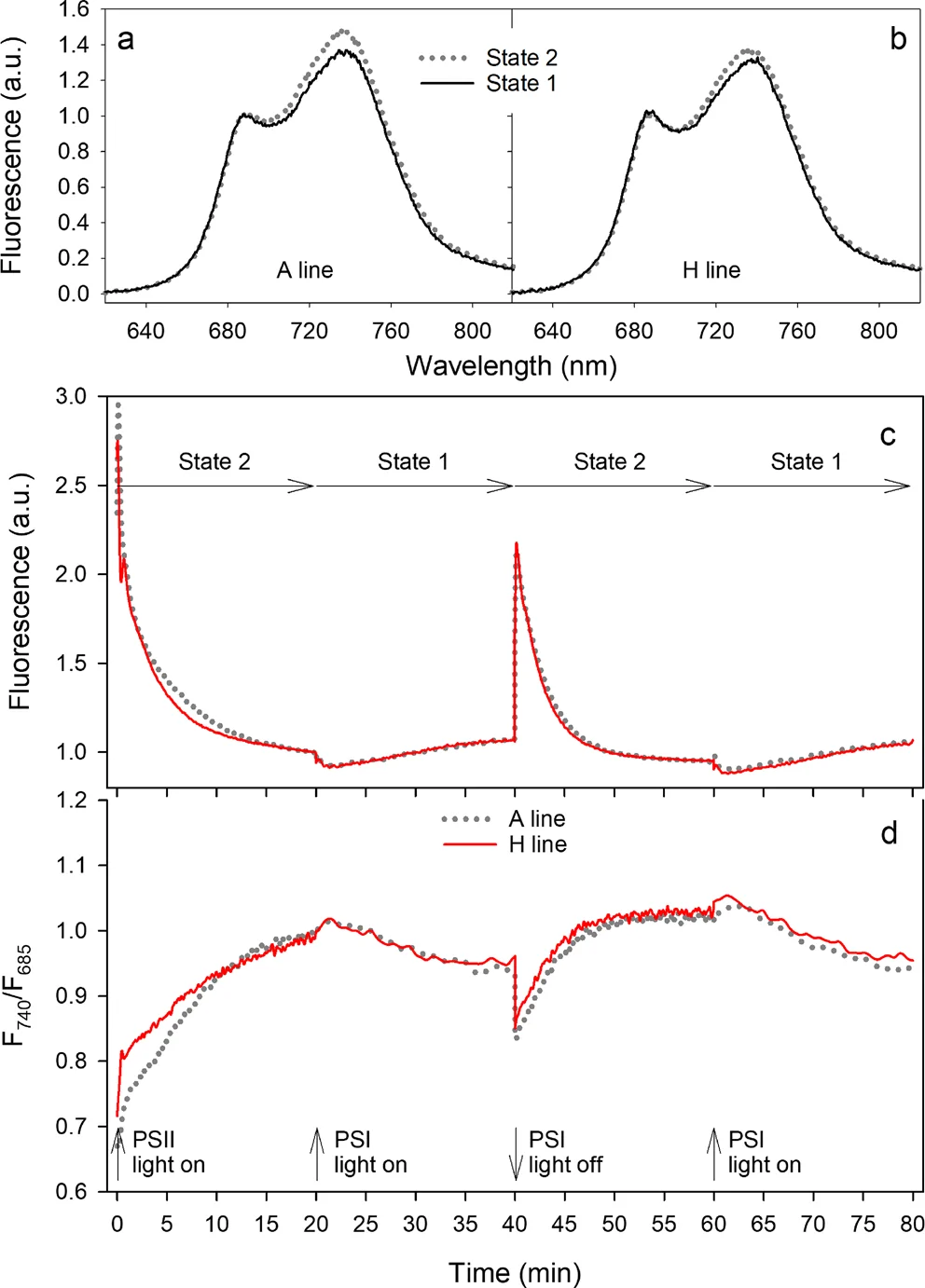
Leaf chlorophyll fluorescence spectroscopic data recorded on non-emitting (**A**) and isoprene-emitting (**H**) tobacco leaves. Fluorescence spectra under 465 nm excitation measured at St2 and St1 for A (**a**) and H lines (**b**), are normalized to the value at the 685 nm band. Kinetics of the total fluorescence intensity (integrated over the whole spectrum) (**c**) and of the F_740_/F_685_ fluorescence ratio (**d**) during state transition; data were normalized to values at 20 min (first St2)

The temporal evolution of the fluorescence bands and of the photosystem‑specific fluorescence intensities shown in Figs. 2, 4, 5 and S4 was filtered using cubic smoothing splines (MATLAB function *csaps*) with a smoothing parameter of 0.01, applied along the time axis to reduce high‑frequency noise while preserving physiologically relevant temporal trends.

## Results

### Pulse amplitude-modulated imaging fluorimetry

The kinetics of the ChlF during state transition as evaluated by Imaging-PAM measurements for the isoprene-emitting (H) and non-emitting (A) tobacco plants are reported in Fig. 1. When both H and A lines reached St2, switching on the FR light induced a fast decrease of ChlF followed by a slower increase typical of the St2→St1 transition. Removal of PSI light led to a steep rise of ChlF reaching a maximum within about 20 s, and then an exponential decay to reach a new St2. The maximum fluorescence $F_{m1}^\prime$ was higher than $F_{m2}^\prime$.

The area-of-interest (AOI) on the same leaf that was not irradiated by the PSI light (control, Fig. S1), showed that once the ChlF reached the steady state it did not change further, and the photosystems remained in St2 (Fig. S3, $F_{m1}^\prime = F_{m2}^\prime$).

The extent of state transition (qT), the maximum quantum yield of PSII photochemistry, the photochemical efficiency of PSII, the nonphotochemical quenching and the fraction of open PSII reaction centres (qL) determined by the Imaging PAM ChlF acquisitions are reported in Table 1.

**Table 1:** Extent of state transition (qT), maximum quantum yield of PSII photochemistry (F_v_/F_m_), photochemical efficiency of PSII (Φ_PSII_), the nonphotochemical quenching (NPQ) and the fraction of open PSII reaction centres (qL) determined by the Imaging PAM ChlF acquisitions. Mean ± SD *n* = 4*

Line	qT (%)	F_v_/F_m_	State	Φ_PSII_	NPQ	qL
A	5.4 ± 0.4	0.759 ± 0.042	St1	0.621 ± 0.045	0.217 ± 0.071	0.84 ± 0.02
	*(1.1±0.8)*^*#*^		St2	0.619 ± 0.042	0.272 ± 0.080	0.86 ± 0.01
H	5.2 ± 1.0	0.746 ± 0.024	St1	0.635 ± 0.048	0.268 ± 0.055	0.82 ± 0.05
	*(0.9±1.3)*^*#*^		St2	0.629 ± 0.043	0.315 ± 0.061	0.85 ± 0.03

*No statistically significant differences between A and H lines were observed for all the parameters. Within the same line, Φ_PSII_, NPQ and qL were not different when measured in St1 and St2. ^#^These values refer to the calculation of qT on the control AOI on leaves unirradiated by FR light, in which no ST is expected to occur

When qT was determined in the leaf areas unirradiated by the PSI FR light, it resulted equal to 1.1 ± 0.8 and 0.9 ± 1.3 for the A and H lines, respectively, as reported in Table 1 for comparison to the qT of the FR irradiated samples. As expected, the low qT values and elevated CVs demonstrate that qT was virtually absent and oscillated around zero.

### Chlorophyll fluorescence spectroscopy

Whole chlorophyll fluorescence spectra were recorded at high frequency during the dark to light transient and the following induction of state transition. Examples of the chlorophyll fluorescence spectra under blue excitation recorded at St2 and St1 on the A and H tobacco lines are reported in Fig. 2a and b, respectively. Whereas, Fig. 2c reports the total Chl fluorescence intensity (the integral over the whole spectral range), and Fig. 2d shows kinetics of the F_740_/F_685_ fluorescence ratio, when repeatedly switching between St2 and St1.

The two tobacco lines showed similar fluorescence spectral shape and intensity temporal change. As expected, the F_740_ nm band relative to the F_685_ nm band was slightly higher in St2 than in St1, due to the different allocation of the excitation absorbed energy between PSII and PSI (Fig. 2a,b). However, because of the overlapping of PSI and PSII contribution to F_740_, the fluorescence analysis of F_740_/F_685_ cannot provide information on the ChlF contribution by the single photosystems. Kinetics of the ChlF integrated over the whole fluorescence spectrum were similar to those recorded by the Imaging-PAM fluorimeter and shown in Fig. 1. In fact, adding of PSI light caused a drop of the signal, followed by a slow increase to the St1 plateau. Whereas, removing PSI light induced a rapid increase of ChlF and a subsequent exponential decrease to the steady value of St2 (Fig. 2c). The F_740_/F_685_ fluorescence ratio followed a behaviour exactly specular to the ChlF intensity change (Fig. 2d).

### Retrieval of PSI and PSII fluorescence spectral contributions

**Fig. 3:**
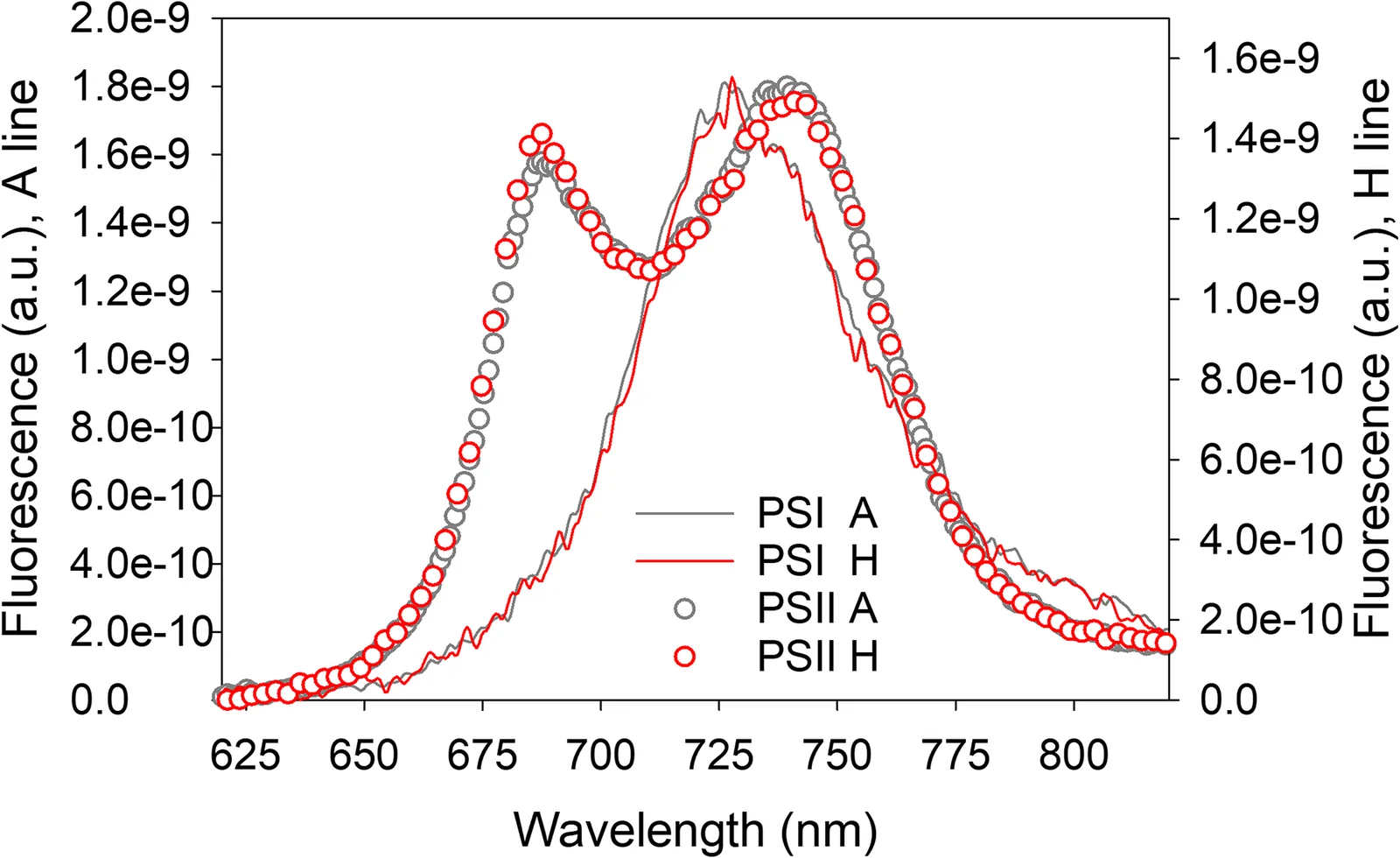
PSI and PSII fluorescence emission spectra, excited at 465 nm, of the A and H *N. tabacum* lines retrieved by the PCA method applied during the St1→St2 longer phase (43.4-60 min) of the post-FR fluorescence transient. Spectra were normalized to the maximum value integrated over a 10 nm waveband around the peak wavelength (730 nm and 735 nm for PSI and PSII, respectively)

The algorithm for retrieving PSI and PSII spectral fluorescence components (Palombi et al. 2011) was applied to our dataset as detailed in the Appendix A. Briefly, the analysis of the ChlF emission spectra recorded during the selected time interval of the St1 → St2 post-FR kinetics provided the PSI and PSII F emission spectra reported in Fig. 3, for the two tobacco lines.

The PSI fluorescence band peaked at about 730 nm, while PSII showed maxima at 687 and 735 nm. No difference in both PSI and PSII spectral shape was observed between the two tobacco lines. The spectral shape of the retrieved PSI and PSII emission are specific of the leaf under measurement because they include the partial re-absorption of the F_685_ band, depending on the specific Chl content of the leaves under investigation. This effect is less evident in the far-red spectral range, and indeed the shape of the retrieved PSI fluorescence spectrum was very similar to that previously measured on isolated PSI-LHI isolated complexes from *N. tabacum* (Chukhutsina et al. 2020).

### State transition PSI and PSII fluorescence kinetics

**Fig. 4:**
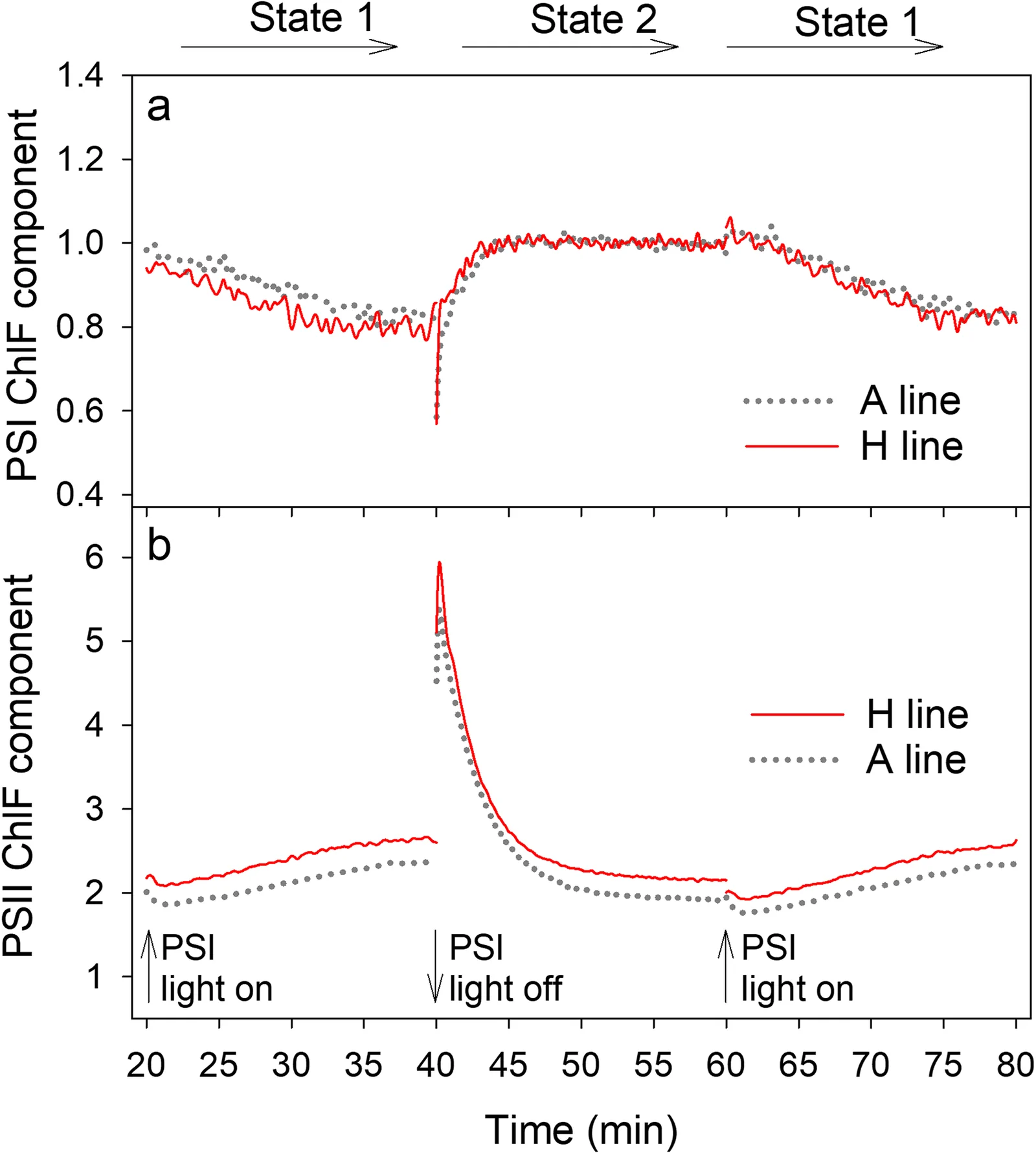
Temporal evolution of the PSI (**a**) and PSII (**b**) contributions to total ChlF during ST for A (grey dots) and H (red lines) *N. tabacum* line leaves. The time scale is the same of Fig. 2d, referring as origin to the start of irradiation with blue light in dark-adapted leaves

The PSI and PSII fluorescence spectra were used to better characterize the ST kinetics, that is, the temporal evolution of the PSI and PSII contributions (the $${k_I}$$ and $$ {k_{II}}$$ coefficients of Eq. 2) for A and H tobacco lines (Fig. 4).

**Fig. 5:**
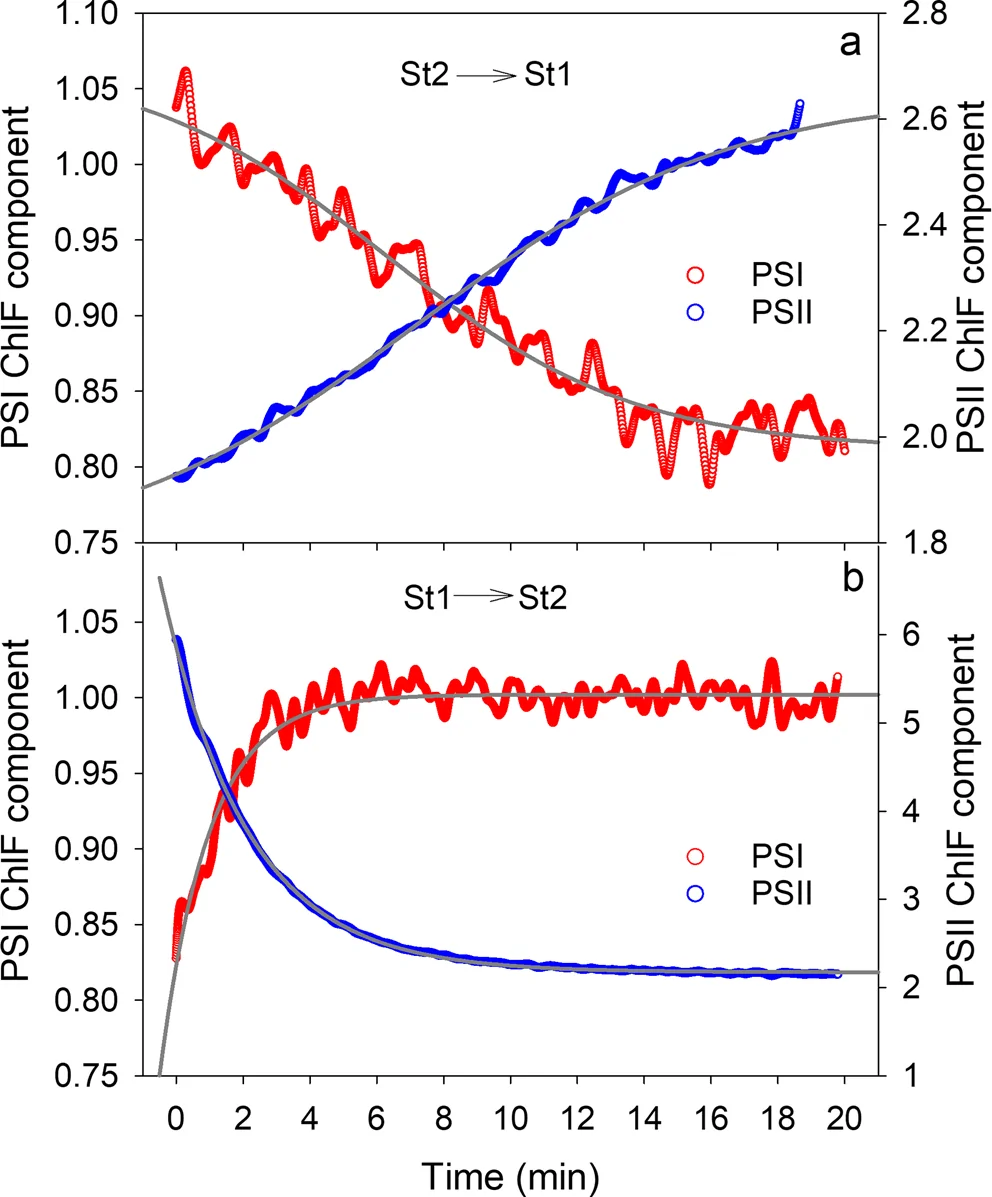
Examples of curve fitting of the PSI and PSII ChlF components kinetics during St2→St1 (**a**) and St1→St2 (**b**) transitions of a *N. tabacum* H line leaf. In (**a**), sigmoid curves with equation f = y0+a/(1+exp(-(x-x0)/b)) were used. r^2^ of fitting was 0.961 and 0.997 for PSI and PSII respectively. In (**b**), a rising exponential curve with equation f = y0+a*(1-exp(-b*x)) was used for PSI (r^2^ = 0.907) and a decreasing exponential curve with equation f = y0+a*exp(-b*x) was used for PSII (r^2^ = 0.999)

During the St2→St1 transition, the PSI and PSII ChlF components were characterized by an opposite sigmoidal behaviour as determined by curve fitting of data. In Fig. 5, an example of the curve fitting for the H line is reported. Similar results were obtained for the A line (Fig. S4). The PSI component decreased with time until St1 was reached. Concurrently, the PSII component increased to a plateau (Fig. 5a). When the PSI light was removed, the PSI component rapidly increased during the first 4–5 min and then remained constant. Concurrently, the PSII component decreased until St2 was reached (Fig. 5b). During this St1→St2 transition, data were well fitted by increasing and decreasing exponential curves for the PSI and PSII components, respectively.

For each photosystem component and state transition, data fitting was of high quality, being r^2^ ranging from 0.91 to 0.99.

**Fig. 6:**
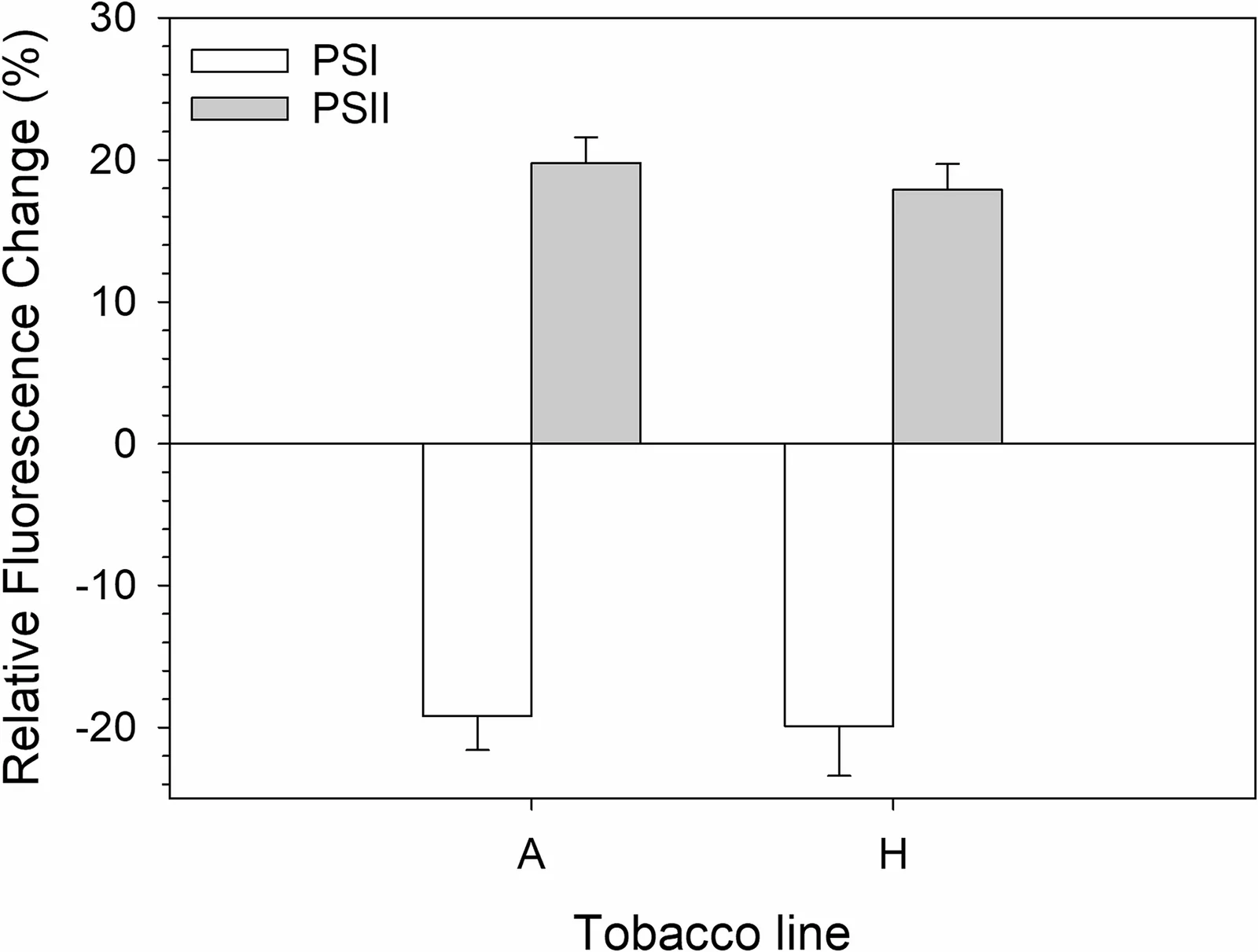
Relative changes in the photosystem ChlF components, (ChlF_1_-ChlF_2_)/ChlF_2_, during the St2→St1 transition for A and H leaves of *N. tabacum* lines. Mean values (±SD) were determined on 3 replicates

From this analysis, the rates and the extent of state transition were determined from the changes in the PSI and PSII fluorescence components and reported in Table 2 and Fig. 6, respectively.

**Table 2:** Rate expressed as the half time, t_1/2_ (min), of the change in the PSI and PSII fluorescence components for the A and H tobacco lines. Fitting estimated time, t_∞_ (min), to reach the steady state level. Mean ± SD *n* = 3,4*

	t_1/2_ (min)			t_∞_ (min)	
	A	H		A	H
St2→St1					
PSI	8.42 ± 0.55 a	6.86 ± 0.49 b		24.0 ± 2.0 a	21.0 ± 1.7 b
PSII	9.14 ± 0.67 a	7.53 ± 1.06 b		29.1 ± 3.2 a	23.9 ± 3.2 b
St1→St2					
PSI	1.04 ± 0.06	0.98 ± 0.05		6.9 ± 0.2	6.6 ± 0.1
PSII	1.88 ± 0.15	1.73 ± 0.13		13.7 ± 1.7	12.9 ± 1.8

*For each parameter and photosystem components, significant differences in values between A and H lines, are marked by different lower-case letters. Statistical comparisons were performed by the Welch’s t-test

The St2→St1 transition was 1.2 times faster in the H line than in the A line, as evaluated by the rate of decrease and increase in the PSI and PSII fluorescence, respectively. Consequently, the time required to reach St1 was longer in the A line than in the H line (24 vs 21 min for PSI and 29 vs 24 min for PSII).

During the St1 to St2 transition, the PSI component reached the steady state within about 7 min on both lines, more than 3 times faster than during the St2 to St1 transition. The transition rate was also similar for the A and H tobacco lines, with t_1/2_ of the order of 1 min. The PSII component during the St1 to St2 transition reached the steady state within approximately 15 min, with a half-time of about 1.8 min. This behaviour is not attributable to ST alone, because PSII fluorescence in this phase is also influenced by non‑photochemical quenching associated with plastoquinone pool reoxidation. The extent of the state transition was determined as the change of the PSI and PSII ChlF components relative to their values at St2. A 20% reduction of the PSI component was matched by a similar increase in the PSII component in both lines (Fig. 6). When the FR PSI light was removed, the state transition was reverted reaching a new St2 with PSI and PSII components almost identical to those present at the previous St2 in both lines.

## Discussion

### Rate of state transition

The state transition rates derived from the kinetics of the PCA-retrieved PSI fluorescence component (Fig. 5) were markedly faster than those obtained with conventional approaches, particularly for the St1→St2 transition, which exhibited a t₁/₂ of approximately 1 min (Table 2).

Earlier work by McCormac et al. (1994) reported considerably slower dynamics in *Spirodela oligorrhiza*, with t₁/₂ values of about 10 min for the St2→St1 transition and 3 min for the St1→St2 transition, based on discrete temporal sampling points of the 77 K F_735_/F_685_ fluorescence ratio.

Consistent with those findings, our exponential fitting of the room temperature F_740_/F_685_ ratio in the tobacco A line yielded average t₁/₂ values of 10.95 ± 2.46 min (St2→St1) and 2.57 ± 0.23 min (St1→St2) (Fig. 2d). In *A. thaliana*, similar St1→St2 rates (t₁/₂ ≈ 149s) have been reported using PAM ChlF measurements (Damkjær et al. 2009).

We attribute the substantially shorter St1→St2 rates obtained in our study to the complete isolation of the PSI fluorescence component, a condition not achieved when using either F_far-red_/F_red_ fluorescence ratios or PAM-based measurements. Even at 77 K, PSII emission contributes non negligibly to the far red region, accounting for roughly 10–20% of the signal (Strasser and Butler 1977b; Terashima et al. 2025). Likewise, PAM detected fluorescence inherently contains both PSII and PSI components. The superposition of these emissions—each with distinct kinetic behaviors—compromises the direct association between the measured temporal changes and the actual state transition process.

These considerations indicate that separating PSI and PSII fluorescence components, combined with high frequency kinetic detection, enables a more accurate determination of ST rates.

It is known that ST in the photosynthetic organisms is controlled by the phosphorylation and dephosphorylation of light harvesting complex II (LHCII) proteins (Allen 2003). Early studies showed a correspondence between the phosphorylation and dephosphorylation rates and the rates of the St1→St2 and St2→St1 transitions, respectively, determined by the analysis of the 77 K ChlF ratio (McCormac et al. 1994). In that study, half-time values for LHCII phosphorylation and dephosphorylation were found to be about 4 and 13 min, respectively. These values align with the rates of changes during ST in the room temperature ChlF ratio (Fig. 2d), but not with those we derived by the kinetics of the PSI and PSII fluorescence contributions (Table 2).

The faster phosphorylation of the Lhcb2 protein observed in *Arabidopsis* during the St1 to St2 transition (30% of the maximal phosphorylation induced by 10 sec of PSII light, Leoni et al. 2013), could be consistent with the rapid increase of PSI fluorescence observed here (Fig. 5b, Table 2). However, there is also evidence that the ST redistribution of absorbed energy between the two photosystems, and the associated ChlF variation, cannot be fully explained by the phosphorylation/dephosphorylation process. Haldrup et al. (2001) found in *Arabidopsis* that the same extent of ST could be achieved with two different levels of phosphorylation: one equal to half the other, suggesting that additional factors besides LHCII phosphorylation could regulate ST (Haldrup et al. 2001). Cutolo et al. (2023) confirmed the significant role of the Lhcb2 phosphorylation in the PSII fluorescence quenching during St1→St2 transition. Yet, it was shown that a relevant component of qT (≈ 40%) was independent from Lhcb2 phosphorylation. On the other hand, it was proved that the Lhcb3 protein that could not be phosphorylated can be involved in the modulation of the ST rate (Damkjær et al. 2009).

During state transitions in vivo, both the PSII to PSI spillover and photosystem light absorption cross-section changes regulate the excitation energy distribution between the two photosystems (Dau and Hansen 1988; Tan et al. 1998), and efficient energy transfer from PSII to PSI has been observed to occur in the PSI–PSII megacomplex (Yokono et al. 2015; Yokono and Akimoto 2018). Terashima et al. (2025) quantified the contribution of spillover to PSI fluorescence using ChlF induction measurements at 77 K. They reported that at F_m_ spillover accounted for 16–28% of the PSI‑detected signal, across various species and growth light conditions. Importantly, the magnitude of spillover depended on the transition state: it was higher in St1 than in St2, consistent with the larger functional antenna size of PSII in St1. This evidence, together with the fact that spillover provides a faster mechanism for balancing excitation between the two photosystems than the modulation of absorption cross‑sections (Tan et al. 1998), explains the more rapid change in PSI fluorescence we observed during the St1→St2 transition compared with the reversed St2→St1 shift (Fig. 5, Table 2).

### Extent of state transition

Often, the extent of state transitions (ST) is expressed as the relative change in F_m_ between St1 and St2 (qT), measured by PAM fluorimetry and attributed to variations in the PSII absorption cross‑section. Using this approach, qT in *A. thaliana* has been reported to be approximately 5–6% (Haldrup et al. 2001) or around 13% (Damkjær et al. 2009; Cutolo et al. 2023). In *N. tabacum*, we obtained qT values of 5.2% and 5.4% for the H and A lines, respectively, using an Imaging‑PAM fluorimeter (Table 1). However, these data cannot reflect the actual fraction of the LHCII antenna that migrates between PSII and PSI during ST, since PAM devices do not resolve the contributions of the two photosystems. Even at the F_m_ level, PSI fluorescence is not negligible, and its relative contribution increases in St2. Consequently, qT values derived from PAM measurements underestimate the true change in the PSII absorption cross‑section. For example, assuming that PSI fluorescence corresponds to about 10% of $F_{m1}^\prime$ (Agati et al. 2000) and, based on our results, that PSI fluorescence increases by roughly 20% from St1 to St2, correcting the F_m_ values for these contributions yields qT estimates that are nearly 50% higher than those obtained with the Imaging‑PAM.

A more reliable quantification of ST can be obtained from time‑resolved fluorescence decay‑associated spectra (Bos et al. 2019). This approach allows the separation of total PSII and PSI emissions, enabling qT to be determined independently for each photosystem. In *A. thaliana* leaves, Bos et al. (2019) reported a qT_PSII_ of 0.14 ± 0.01 (14%) when using F_PSII–St1_ as the reference, corresponding to 16% when expressed relative to F_PSII–St2_, and a qT_PSI_ of 0.19 ± 0.01 (19%) when using F_PSI–St2_ as the reference.

Fluorescence excitation spectroscopy at 77 K, specifically measuring the F_735_ emission at St1 and St2, has also been used to assess PSI cross‑section changes during ST (Ruban and Johnson 2009). In *A. thaliana*, this method yielded a qT_PSI_ of approximately 24% when expressed relative to St1, which corresponds to 19% when reported relative to F_PSI–St2_, a value identical to the estimate obtained by Bos et al. (2019) using time‑resolved fluorescence.

It is important to emphasize that comparing qT values across studies requires expressing them relative to the same reference fluorescence state. When this is done, the 18–20% changes in PSI and PSII fluorescence that we observed between St2 and St1 in the A and H tobacco lines align well with the ST amplitudes reported for *A. thaliana* in studies where the two photosystem emissions were experimentally separated (Ruban and Johnson 2009; Bos et al. 2019).

### Is the PCA‑based ST analysis robust to ChlF reabsorption?

The fluorescence spectrum detected outside an intact leaf is not simply the intrinsic emission spectrum of the photosystems. Rather, it reflects the combined effect of: (i) the penetration of the incident excitation light into the tissue, which determines the depth distribution of excited chloroplasts; and (ii) the propagation of the emitted fluorescence photons toward the detector, including reabsorption and scattering within the leaf. Both processes depend on leaf optical properties, including chlorophyll content, thickness, and internal structure. Therefore, the PSI and PSII spectra measured from intact leaves are leaf-specific effective spectra rather than intrinsic spectra of isolated photosystems. The point relevant to the present analysis is whether these optical effects can bias the determination of the temporal evolution of the PSI and PSII fluorescence contributions during ST. This aspect is properly discussed in the Appendix B. In there, it is shown that, if leaf optical properties remain constant during the analysed time window, the combined effect of excitation penetration and fluorescence escape can be represented by a single time-invariant effective optical transfer function. Under this condition, the observed PCA-retrieved photosystem spectral shapes are modified, but the state-transition amplitudes derived from the fitted PSI and PSII contributions remain unchanged.

### Photosystem ChlF versus light absorption cross section changes

**Fig. 7:**
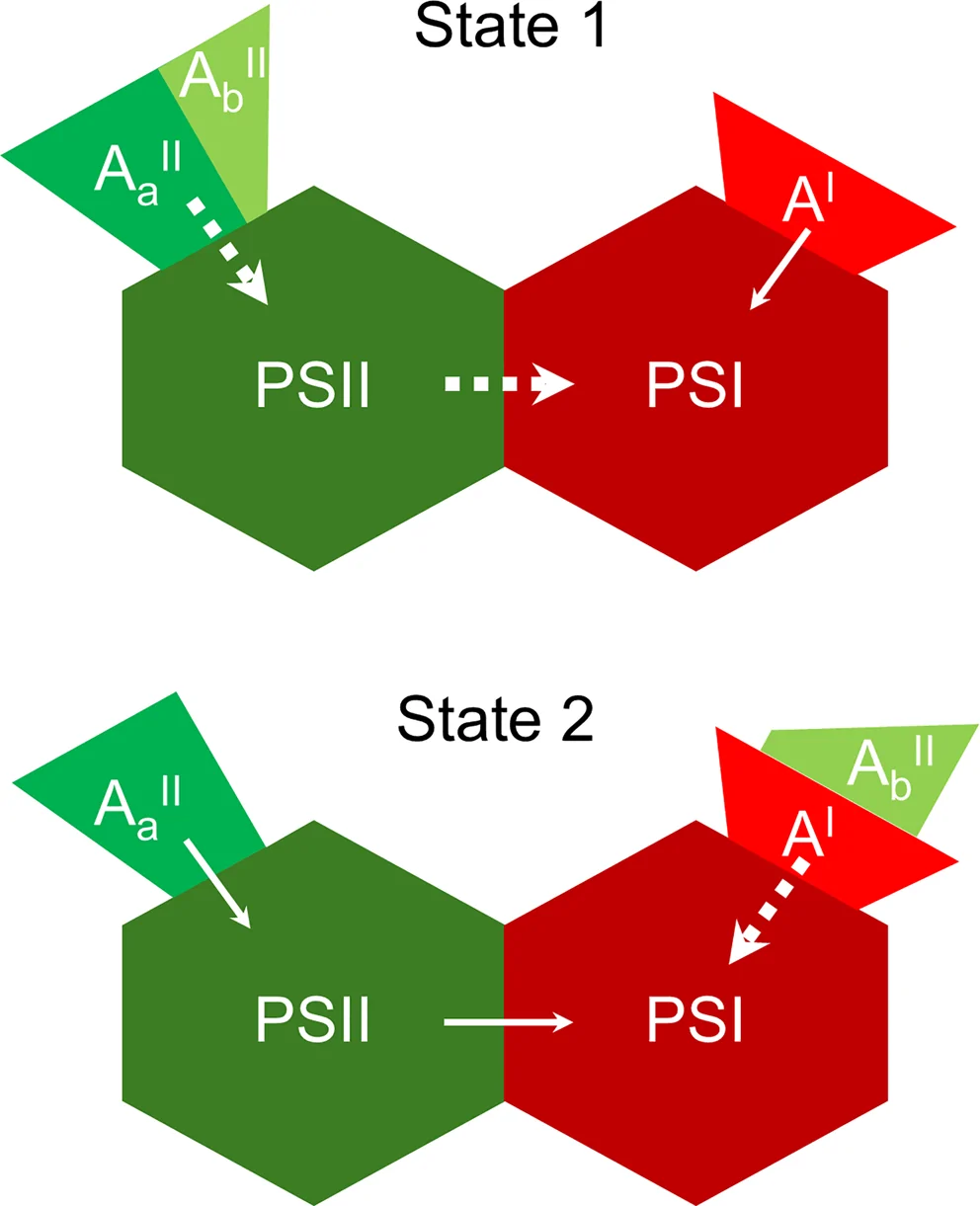
Scheme of the energy transfer between antennas and photosystems at state 1 and state 2. The$$ {A^{II}}$$ PSII antenna is composed by $$A_a^{II}$$ that includes the strongly LHCII-S and moderate LHCII-M bound trimers and $$A_b^{II}$$ corresponding to the loosely bound LHCII-L trimer. This last antenna is the mobile fraction that during St1→St2 detaches from $$ {A^{II}}$$ and attaches to the PSI antenna ($${A^I}$$). The large-size dashed arrows indicate higher energy transfer than the small size solid arrows. The direct PSII→PSI energy transfer represents spillover

As discussed above and in the literature, the magnitude of ST is estimated from the relative changes in the photosystem ChlF during the transition. This variation is typically interpreted as an indicator of the relative modulation of the antenna size of each photosystem. However, whether the observed ChlF changes correspond exactly to the underlying changes in photosystem light absorption cross sections remains an open question. To address this issue, the effect of PSII→PSI spillover during ST must be taken into account. A schematic representation of the antenna‑absorbed energy and its distribution to and between the two photosystems at St1 and St2 is shown in Fig. 7.

In accordance to the early models for the excitation energy distribution in the chloroplast photosynthetic apparatus (Strasser and Butler 1977a, b), we derived a quantitative relationship between the measured photosystem fluorescence changes and the corresponding changes in light absorption cross sections. We explicitly considered that (i) the LHCII PSII antenna is composed by two parts $${A^{II}} = A_a^{II} + A_b^{II}$$, where $$A_a^{II}$$ is related to the stable strongly LHCII-S and moderate LHCII-M bound antennas and $$A_b^{II}$$ indicates the loosely bound LHCII-L antenna that moves between PSII and PSI during ST; (ii) the PSI fluorescence includes an intrinsic term due to excitation delivered from its own antenna ($${A^I}) $$ and an additional term due to spillover from PSII. By using the Strasser and Butler (1977c) formalism, these two terms refer to the $${F_{PSI, \alpha }}$$ and $${F_{PSI,\beta }}$$ fractions, respectively.

The full derivation (Eqs. C.1–C.13) and the definition of all terms of the ChlF dependence on excitation absorbance and distribution are reported in Appendix C.

In summary, we proved that

3 $${{\Delta F_{PSII}^{St1 - St2}} \over {F_{PSII}^{St2}}} = {{A_b^{II}} \over {A_a^{II}}}$$

That is, the relative change of F_PSII_ during ST equals the ratio of the two ${A^{II}}$ parts, corresponding to the ratio of the PSII light absorption cross sections (Eqs. C.5 in Appendix C).

From the measured F_PSII_ of the two tobacco lines it resulted that the $A_b^{II}$ fraction of LHCII moving during ST should be of about 17% of the total ${A^{II}}$.

From the same approach applied to F_PSI_, it was not possible to obtain directly the percentage change in the PSI light absorption cross section during ST, since the derived equation (Eqs. C.9 in Appendix C) containing multiple unknowns is unsolved. However, by using the Terashima et al. (2025) formalism, it was possible to express the ratio between the measured F_PSI_ at St2 and St1 as

4 $${{F_{PSI}^{St2}} \over {F_{PSI}^{St1}}} = {{F_{PSI,\alpha }^{St2}} \over {F_{PSI,\alpha }^{St1}}} \cdot {C_{spillover}} $$

the corresponding ratio between the intrinsic ${F_{PSI, \alpha }}$ contributions, multiplied by a correction factor that reflects the spillover-dependent F_PSI_ relative to the intrinsic one (${F_{PSI,\beta }}/{F_{PSI, \alpha }})$ under the two states (Eq. C.12 in Appendix C).

Terashima et al. (2025) determined the ${F_{PSI, \beta }}$ contributions relative to the total F_PSI_ at St1 and St2 for four different species grown under various light intensities, while accounting for different levels of F_PSII_ contamination in the F_PSI_ signal.

Those measurements were obtained under conditions in which reaction centres were largely in the reduced (closed) form, a situation markedly different from that of the present study, where plastoquinones were predominantly in the oxidized form (qL, Table 1). To derive the ${C_{{\rm{spillover}}}}$ factor appearing in the right-hand side of Eq. (4), we therefore rescaled Terashima’s ${F_{{\rm{PSI}},\beta }}/{F_{{\rm{PSI}},m}}$ values by the ratio between ${F_m}$ and ${F_0}$ (2–2.5), corresponding to the difference in PSII→PSI energy transfer yields originally reported by Strasser and Butler (1977a, c). The resulting ${C_{{\rm{spillover}}}}$ ranged between 0.92 and 0.97. Consequently, ${{F_{PSI,\alpha }^{St2}} \over {F_{PSI,\alpha }^{St1}}}$, that corresponds to the ratio of the PSI light absorption cross sections between St2 and St1, should be within 1.27 and 1.34.

Under this simulation, the PSI absorption cross‑section would increase by about 31% from St1 to St2, a value substantially higher than the 17% change derived for PSII. These findings suggest that the mobile LHCII pool associated with PSI under St2 conditions represents a larger proportion of the PSI antenna than it does of the total PSII antenna. A relative increase in the PSI cross‑section that is 1.6‑times greater than the relative decrease in the PSII cross‑section was previously observed in spinach thylakoids upon phosphorylation induction (Samson and Bruce 1995).

Remarkably, the increase in the PSI absorption cross‑section we report for the St1→St2 transition aligns well with the 30% and 33% increases in functional PSI antenna size measured in *A. thaliana* wild‑type intact thylakoids (Schiphorst et al. 2022) and leaves (Lunde et al. 2000), respectively, based on changes in P700 oxidation. It is also consistent with the 27% fraction of phosphorylated Lhcb2 (P‑Lhcb2) associated with PSI at St2 relative to dark‑adapted (near‑St1) conditions previously determined in *N. tabacum* (Chukhutsina et al. 2020).

### Is isoprene involved in state transition?

Tobacco plants may have experienced mild stress due to the warm weather conditions during the trial. However, plants showed a high maximal yield of PSII (Fv/Fm ≈ 0.75; Table 1), a high efficiency of PSII photochemistry (Φ_PSII_) and a low NPQ (Table 1), indicating that plants could be considered as unstressed, and this condition was observed both in isoprene-emitters and non-emitters. Even at physiological temperatures, isoprene-emitting plants are able to maintain higher photochemical efficiency and higher stabilization of the photosynthetic membranes with respect to their corresponding transgenic isoprene-emitting lines (Pollastri et al. 2014). In the absence of isoprene, the lower content of unsaturated fatty acids in chloroplasts results in reduced thylakoid fluidity (Velikova et al. 2015).

During state transition, phosphorylation of LHCII induces structural rearrangements of the thylakoid membranes (Chuartzman et al. 2008), including changes in grana size and stacking as well as modifications in the interactions between photosystem complexes and their antenna systems (Wood and Johnson 2020). The kinetics of F_PSI_ and F_PSII_ during the St2→St1 transition were faster in the isoprene‑emitting H line than in the non‑emitting A line. This observation is consistent with the role of isoprene in stabilizing thylakoid membrane ultrastructure and fluidity, both conditions being essential for efficient photosynthetic function (Velikova et al. 2015). It is possible that the presence of isoprene in the H tobacco line facilitates the ST excitation energy distribution between PSII and PSI, making faster t_1/2_ and shorter t_∞_, the time to reach steady state PSI and PSII fluorescence during St2 to St1 transition (Table 2). Conversely, no differences between H and A tobacco transgenic lines were appreciated in the PSI fluorescence change during the faster St1→St2 transition. Currently, we cannot explain why isoprene affects the St2→St1 transition but not the reverse process. Possibly, the reason lies in the mechanisms of LHCII phosphorylation and dephosphorylation as well as protein migration that regulate differently the two ST phases (Rantala et al. 2020). These include: diverse substrate specificities for the kinase and phosphatase enzymes (Wei et al. 2015; Liu et al. 2016); the functional requirement of a rapid phosphorylation-mediated transition to St2 as the plant response to light intensity change (Tikkanen and Aro 2012), non-urgent for the reversed dephosphorylation-mediated transition to St1; faster physical migration of the phosphorylated-LHCII with respect to the dephosphorylated LHCII complexes (Consoli et al. 2005).

Different rates of ST were observed comparing *Arabidopsis* and barley plants and ascribed to a difference in the viscosity of the thylakoid membranes between the two species (Vetoshkina et al. 2024). Therefore, it should be cautioned that the impact of isoprene on ST may be different in different plant species. Finally, we cannot exclude that, during the St1→St2 transition, the larger contribution of spillover compared with the St2→St1 transition could mask potential differences in PSI fluorescence kinetics between H and A lines. Consistently, the extent of state transitions, estimated from the relative changes in PSII and PSI fluorescence contributions, did not differ significantly between the two lines (Fig. 6).

## Conclusions

Although the mechanisms underlying state transitions have been long known, new models that reveal additional layers of complexity continue to be developed and debated. Here, we successfully applied an enhanced fluorescence spectroscopic approach based on PCA‑assisted spectral analysis to intact tobacco leaves on which ST was elicited using lights with selected wavelengths. The spectral separation of PSI and PSII allowed us to extract fluorescence kinetics with greater precision, providing more accurate estimates of both the rates and the extent of the transition compared with earlier methods. In particular:

i) the spectral separation achievable with 77 K fluorescence spectroscopy is incomplete because the PSI and PSII far‑red emission bands partially overlap;
ii) time‑resolved fluorescence decay‑associated spectra provide a powerful means to distinguish PSI and PSII contributions, but the technique is not suitable for time‑course analyses;
iii) PAM‑detected fluorescence, although widely used, contains both PSII and PSI components, so its temporal changes and overall amplitude reflect processes beyond state transitions alone.

The present study proposes a model that further clarifies the relationship between PSI and PSII chlorophyll fluorescence changes and the relative variations in their absorption cross sections during state transition. This aspect was not clearly resolved by previous measurements, and provides new insight into the mechanisms governing the process. By incorporating the contribution of PSII‑to‑PSI spillover, our analysis indicates that the mobile LHCII fraction associated with PSI during the St1→St2 transition leads to a 31% increase in the PSI absorption cross section. Conversely, when the mobile LHCII pool associates with PSII, the resulting change in absorption cross section is limited to 17%. These differences in energy distribution between the two photosystems are likely to have significant physiological implications for photosynthetic performance in dynamic environments, including sunflecks, canopy shade, and high-light stress.

The PCA-supported spectral analysis was applied to investigate the possible involvement of isoprene in the ST of tobacco leaves. Our results indicate that isoprene promoted a faster redistribution of excitation energy between PSI and PSII during the St2→St1 transition, likely by maintaining optimal thylakoid ultrastructure and fluidity. It would be interesting to further investigate this effect in other isoprene-emitting transgenic species (*Arabidopsis thaliana*), and for example by exposing emitters to light or temperature fluctuations, as experienced in nature, or by providing to plants pharmacological doses of exogenous isoprene.

The only limitation for the proper applicability of the method is the requirement that the optical properties of the leaf remain constant during the measurements. This condition is easily fulfilled by using excitation wavelengths that do not induce chloroplast movement or, when blue light is used, by allowing the movement to reach its steady distribution before starting the state‑transition measurements. With this precaution, our approach provides a new tool for analysing chlorophyll fluorescence emission spectra that can be used to investigate the dynamics of energy distribution between PSI and PSII in vivo, directly at physiological temperatures. This may represent a complementary tool to explore how environmental factors and stress conditions influence photosynthetic performance.

## Electronic supplementary material

Below is the link to the electronic supplementary material.

Supplementary Material 1

## Figures and Tables

**Fig. 8:**
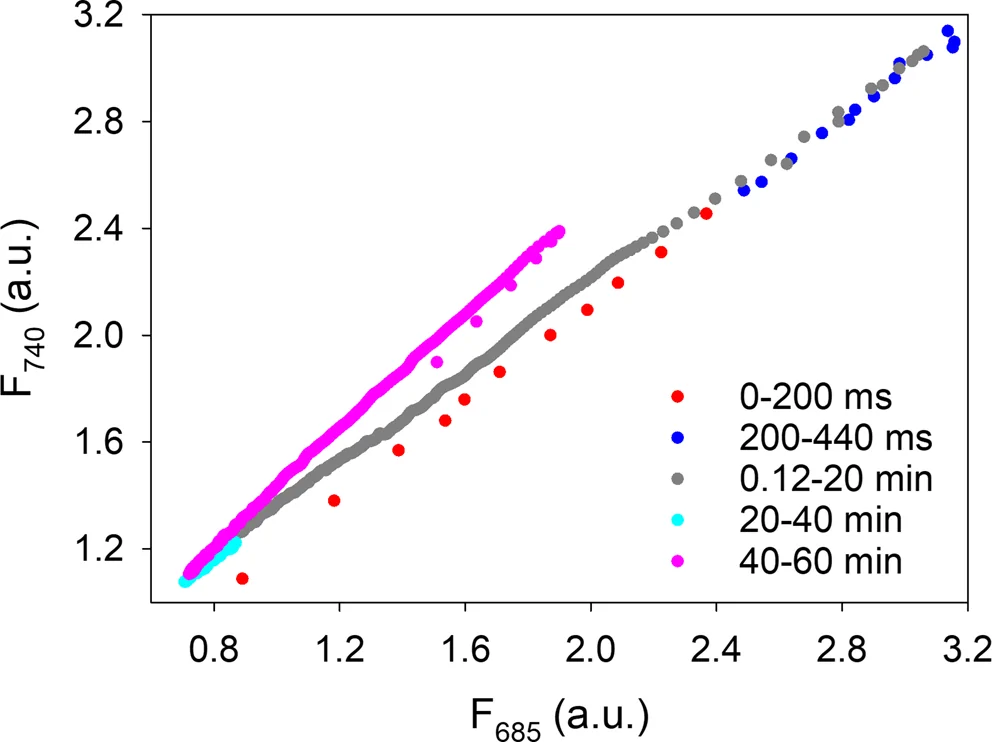
F_740_ vs F_685_ during different phases of the Chl fluorescence induction curves: the initial fast rising of the Kautsky kinetics OI photochemical phase (red dots, 0–200 ms); a second rising phase, the thermal phase (blue dots, 200–440 ms), and the decrease phase of the Kautsky kinetics reaching a St2 steady level (grey dots, 0.12–20 min); the St2 to St1 transition (cyan dots, 20–40 min); the post-FR phase from St1 to St2 (magenta dots, 40–60 min). The plot of the last temporal phase (60–80 min) was overlapped to the first St2 to St1 transition (cyan dots) and it is not reported for clarity. Data refer to the acquisitions on a tobacco A line leaf

**Fig. 9:**
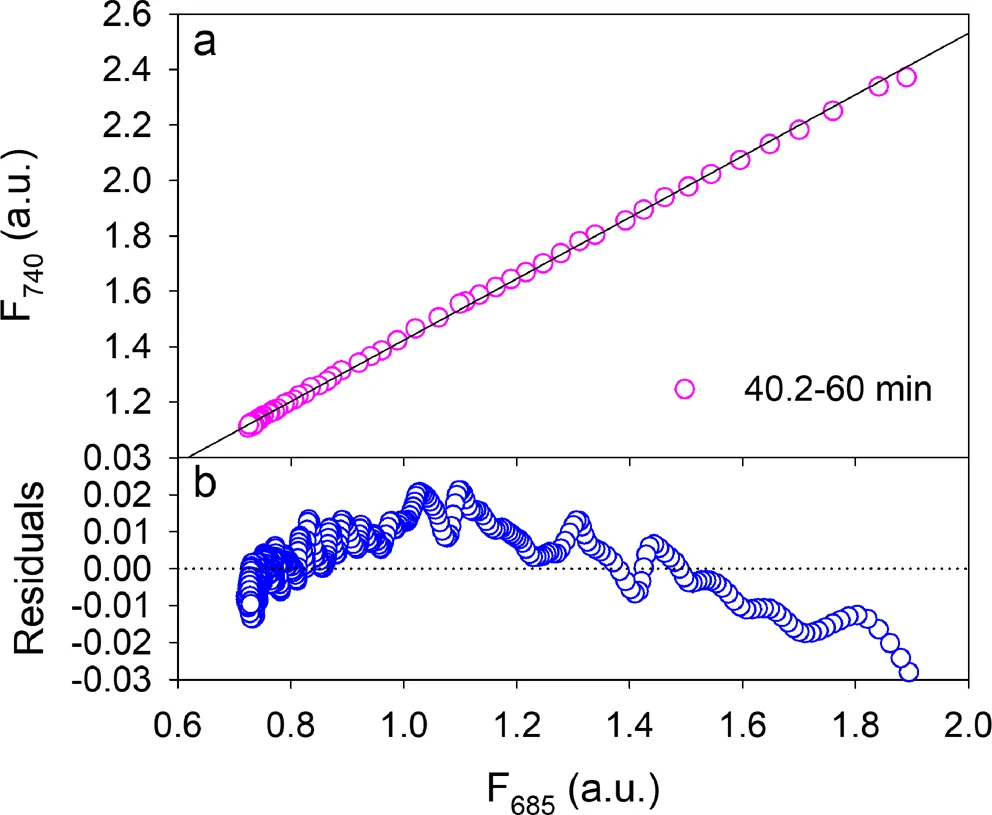
Analysis of the F_740_ versus F_685_ data during the St1→St2 post-FR phase (40.2–60 min, excluding the first 0.2-min rising) reported in Fig. 8. (**a**) Linear regression with fitting equation y = 0.3154+1.1079x (r^2^ = 0.9993); (**b**) distribution of residuals as function of F_685_. Data refer to the acquisitions on a tobacco a line leaf

**Fig. 10:**
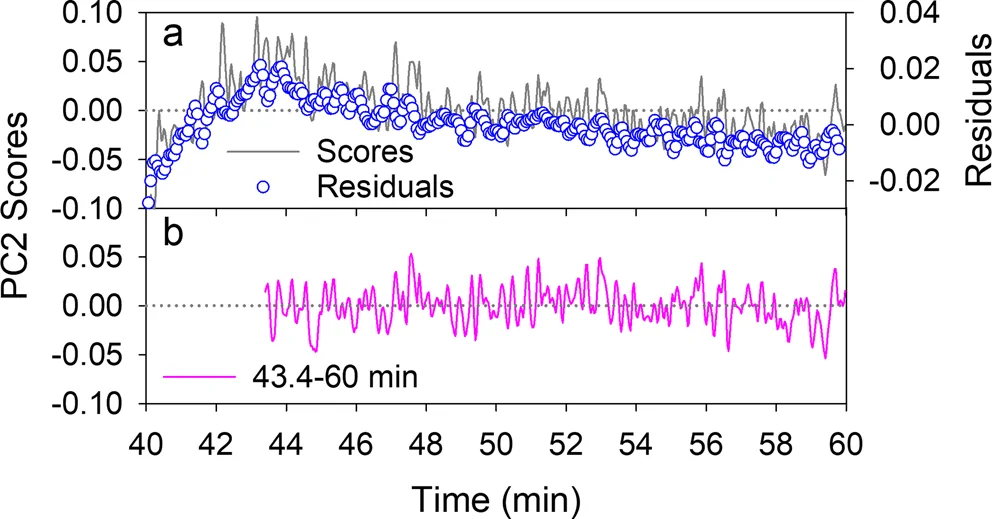
Temporal evolution of PC2 scores for the entire 40–60 min interval (**a**) and for the selected interval starting at 3.4 min from the beginning of the St1→St2 transition (**b**). In (**a**), the kinetics of residuals derived by the linear fitting of the F_740_ vs F_685_ plot of Fig. 9a is also reported (blue dots). Data refer to the acquisitions on a tobacco A line leaf

**Fig. 11:**
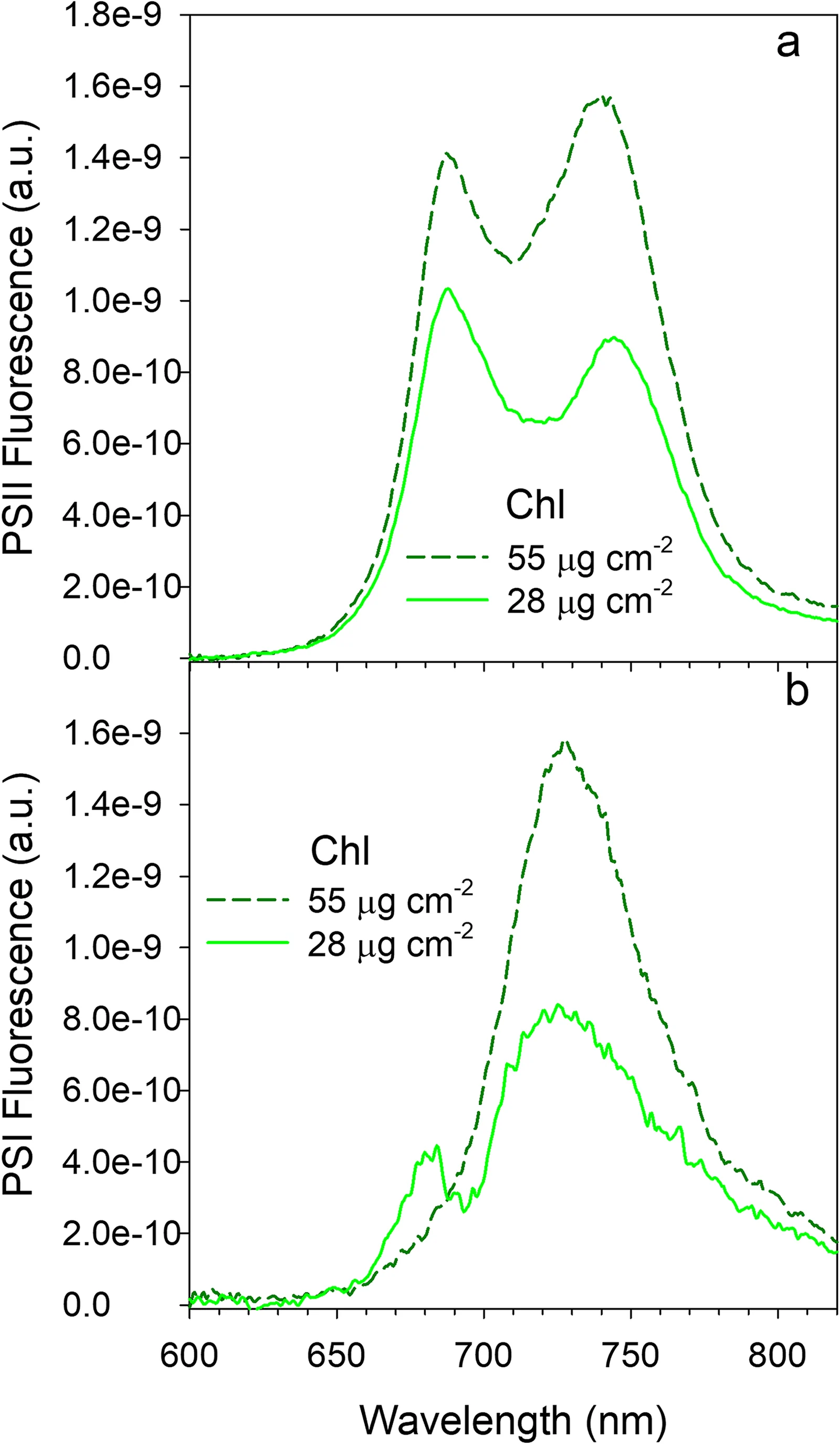
Average PSI and PSII fluorescence emission spectra retrieved by the PCA method from *N. tabacum* leaves with lower (solid light green) and higher (dashed dark green) Chl content, *n* = 3

**Fig. 12:**
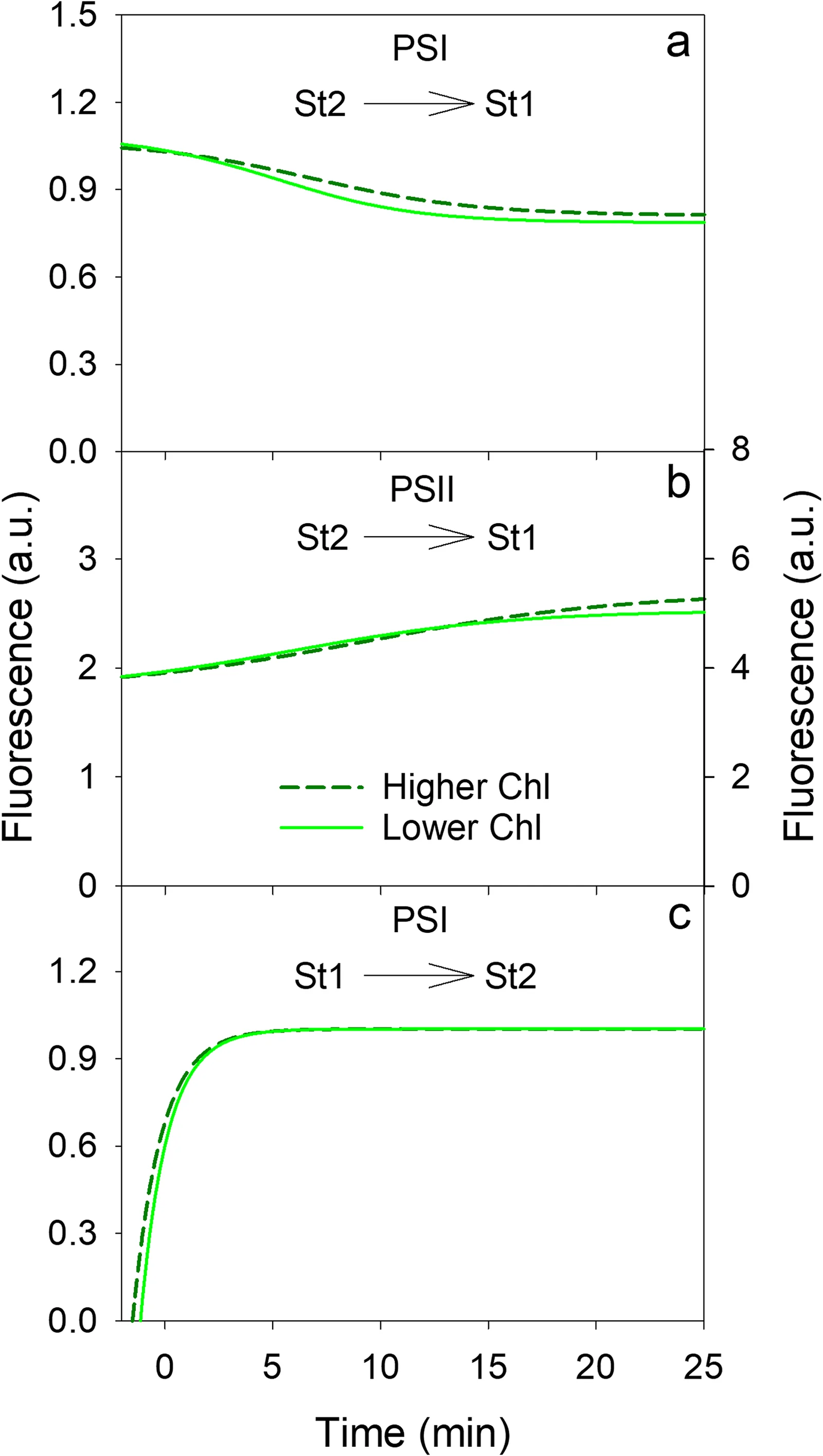
Average fitting curves (sigmoidal in **a** and **b**, exponential in **c**) of the PSI and PSII ChlF components kinetics during St2→St1 (**a, b**) and St1→St2 (**c**) transitions of *N. tabacum* leaves with lower (solid light green) and higher (dashed dark green) Chl content, *n* = 3

## Data Availability

Main data are contained within the article. Additional data will be made available from the authors upon request.

## Appendix A. The algorithm for retrieving PSI and PSII spectral fluorescence components

As explained above, the previously developed algorithm to retrieve the PSI and PSII spectral fluorescence components needs ChlF kinetics datasets in which the PSI contribution is constant and the PSII contribution changes in intensity but not in shape (Palombi et al 2011). These conditions were met during the initial phase of the Kautsky kinetics (origin-inflection (OI) points), when a linear relationship between the far-red and the red ChlF bands was observed (Palombi et al 2011). In the present study, the linearity in the first phase of the F_740_ versus F_685_ plot, the photochemical phase, was limited to a transient of about 200 ms, as seen in Figs. 8 and S5 for tobacco leaves of the A and H lines, respectively. The second fast rising phase (200-440 ms), known as the thermal phase, already presented a change in the slope of the F_740_ vs F_685_ plot (Moise and Moya 2004; Palombi et al 2011).

In this phase, the onset of a PSII light‑adapted charge‑separated state together with protein conformational adjustments (Sipka et al 2021; Garab et al 2023) is expected to introduce additional sources of variability in the PSII fluorescence signal. As a consequence, the PSII emission may not preserve a stable spectral shape, making the application of the retrieving algorithm unreliable under these conditions.

Because of the limited frequency of our detection system, only few spectra (10-11) could be acquired during the first initial ChlF transient, which made results noisier than expected, even using, as done before (Palombi et al 2011), repeated multi-acquisitions of short initial fluorescence transients.

We looked for different temporal phases in the measured ChlF datasets for which the contribution of the two photosystems did not covary.

The time intervals corresponding to non-linear relationships between the two ChlF bands were excluded because here at least two distinct variable fluorescence contributions are involved. The kinetics relative to the two St2 to St1 transitions were also excluded because in this region small changes (around 15%) in the F_740_ and F_685_ (low dynamics) occurred (Fig. 8).

The F_740_ versus F_685_ data during the St1→St2 post-FR phase (excluding the first 0.2-min rising) were well fitted by a linear regression (r^2^ = 0.999) (Fig. 9a), however the residuals presented a non-random distribution around the zero line (Fig. 9b) suggesting the presence of a non-linear pattern.

Applying the PCA to the ChlF spectra acquired during the whole post-FR phase, it resulted that the PC1 described 89.81% of the data variance, while the PC2 described 0.13% of the data variance. Consistently with these values, Bartlett’s test confirmed that only the first principal component was statistically significant (*p* < 1 × 10^−4^), while PC2 was not significant (*p* = 0.75).

Although the PC2 explained a small fraction of the total variance, its scores exhibited a systematic trend over time (Fig. 10a), and therefore its contribution could not be negligible. The temporal evolution of the PC2 scores showed a maximum and a trend inversion around 3-4 min from the beginning of the ST. Actually, as expected, it corresponded exactly, to the kinetics of the residues derived by the linear fitting of the F_740_ vs F_685_ plot (Fig. 10a), indicating the presence of a non-linear relationship between F_740_ and F_685_.

Incremental removal of the initial spectral data (in 100-point steps) revealed the widest temporal range free from systematic trends in the PC2 scores (Fig. 10b).

Accordingly, the range from 43.4 to 60 min, was chosen to apply the photosystem fluorescence components retrieval algorithm. Consisting of approximately 5000 spectra and 35% relative changes in the F_740_ and F_685_, it ensured robust statistical validity. In this phase, the spectral shape was substantially stabilized, with PC2 reduced to minor contributions and the single PC1 as dominant source of variance. As an additional objective check for residual temporal structure, we applied the Ljung–Box portmanteau test to the PC2 score time series, which evaluates the null hypothesis of no serial autocorrelation up to a specified lag order (Ljung and Box 1978). With spectra acquired at 5 Hz (Δt = 0.2 s), we tested lag horizons corresponding to 4, 10, 20, 40, and 60 s. When applied to PC2 scores over the full time window (40.0–60 min), the test returned extremely small p‑values (p < 10^−^^6^), indicating time‑correlated residual variance and thus violation of the one‑dimensional variance assumption required for the PSI and PSII spectral retrieval. In contrast, for the selected reduced interval (43.4–60 min), the test did not reject the null hypothesis at any tested horizon (p = 0.313, 0.166, 0.486, 0.234, and 0.413 for 4, 10, 20, 40, and 60 s, respectively), supporting the absence of systematic temporal dependence in PC2 and independently validating the chosen window as consistent with a variance structure dominated by PC1.

To assess the robustness of the retrieval procedure, we performed a sensitivity analysis by varying independently the start and end times of the selected interval and repeating the PCA-based retrieval and evaluation of the PSII and PSI contributions to Chl fluorescence during ST for each resulting time window. Then, we recalculated, for each interval, ST extent for the two PSs, namely qT_PSI_, qT_PSII_, and the PSII fluorescence ratio between 740 nm and 685 nm bands (as index of the PSII fluorescence spectral shape).

First, the starting time was shifted from 43.4 min back to 42.4 min in 2-s steps, thereby intentionally including part of the earlier phase in which a second structured component is more likely. Across these intervals, the CV was 2.7% for qT_PSI_, 0.9% for qT_PSII_, and 0.7% for the PSII F_740_/F_685_ ratio. When the starting time was instead shifted forward from 43.4 to 44.4 min, again in 2-s steps, the corresponding CVs were 1.6%, 0.8%, and 0.4%, respectively. In a second analysis, the starting time was kept fixed at 43.4 min while the end time was reduced from 60 min to 55 min in 10-s steps. Under these conditions, the CV remained limited, namely 1.2% for qT_PSI_, 1.9% for qT_PSII_, and 0.3% for the PSII F_740_/F_685_ ratio. These results show that, although the retrieval method is inherently dependent on the selected time interval, the estimated parameters are only weakly affected by substantial changes in the interval boundaries. Notably, the variability introduced by interval selection was smaller than that observed among biological replicates, supporting the robustness of the method when the analysis window is selected according to the objective criteria described above.

## Appendix B. Chlorophyll fluorescence reabsorption and robustness of the state transition analysis

This Appendix explains because the spectral change of the PSI and PSII fluorescence components due to ChlF reabsorption inside the leaf does not affect the results of the PCA-based analysis of ST.

### Effective optical transfer function

Let ${\rm{Chl}}{{\rm{F}}_i}\left( {t,\lambda } \right)$ denote the inner leaf fluorescence signal emitted at the chloroplast level in the absence of optical filtering by the surrounding tissue. During the selected time interval, the signal is assumed to satisfy the one-component condition required for the spectral retrieval, namely

B.1 $$\matrix{ {} & {{\rm{Chl}}{{\rm{F}}_i}\left( {t,\lambda } \right) = {h_c}\,{\rm{PS}}{{\rm{I}}_i}\left( \lambda \right) + g\left( t \right)\,{\rm{PSI}}{{\rm{I}}_i}\left( \lambda \right),}\cr } $$

where $${\rm{PS}}{{\rm{I}}_i}\left( \lambda \right) $$ and $${\rm{PSI}}{{\rm{I}}_i}\left( \lambda \right)$$ are the intrinsic PSI and PSII spectral shapes, $${h_c} $$ is the time-independent PSI contribution, and $$g\left( t \right) $$ is the time-dependent PSII contribution.

In an intact leaf, the detected fluorescence depends both on where excitation is generated within the tissue and on the wavelength-dependent probability that emitted photons escape the leaf toward the detector. After averaging over the illuminated leaf volume, these two effects can be represented by an effective optical transfer function ${\rm{\Omega }}\left( \lambda \right)$, which includes both the excitation and the emission pathways. The detected spectrum can therefore be written as

B.2 $$\matrix{ {} & {{\rm{ChlF}}\left( {t,\lambda } \right) = {\rm{\Omega }}\left( \lambda \right)\,{\rm{Chl}}{{\rm{F}}_i}\left( {t,\lambda } \right).} & {} & {} \cr } $$

Substituting Eq. (B.1) into Eq. (B.2) gives

B.3 $$\matrix{ {} & {{\rm{ChlF}}\left( {t,\lambda } \right) = {\rm{\Omega }}\left( \lambda \right)\left[ {{h_c}\,{\rm{PS}}{{\rm{I}}_i}\left( \lambda \right) + g\left( t \right)\,{\rm{PSI}}{{\rm{I}}_i}\left( \lambda \right)} \right]} & {} & {} \cr } $$

and therefore

B.4 $$\matrix{ {} & {{\rm{ChlF}}\left( {t,\lambda } \right) = {h_c}\,{\rm{\Omega }}\left( \lambda \right)\,{\rm{PS}}{{\rm{I}}_i}\left( \lambda \right) + g\left( t \right)\,{\rm{\Omega }}\left( \lambda \right)\,{\rm{PSI}}{{\rm{I}}_i}\left( \lambda \right).} & {} & {} \cr } $$

Defining the observed, leaf-specific photosystem spectra as

B.5 $$\matrix{ {} & {{\rm{PSI}}\left( \lambda \right) = {\rm{\Omega }}\left( \lambda \right)\,{\rm{PS}}{{\rm{I}}_i}\left( \lambda \right),} & {} & {} \cr } $$

B.6 $$\matrix{ {} & {{\rm{PSII}}\left( \lambda \right) = {\rm{\Omega }}\left( \lambda \right)\,{\rm{PSI}}{{\rm{I}}_i}\left( \lambda \right),} & {} & {} \cr } $$

Eq. (B.4) becomes

B.7 $$\matrix{ {} & {{\rm{ChlF}}\left( {t,\lambda } \right) = {h_c}\,{\rm{PSI}}\left( \lambda \right) + g\left( t \right)\,{\rm{PSII}}\left( \lambda \right).} & {} & {} \cr } $$

Thus, the combined optical effects of excitation penetration and fluorescence escape do not change the mathematical structure of the retrieval algorithm. They only transform the intrinsic photosystem emissions into the leaf-specific spectra effectively observed from the intact leaf. If ${\rm{\Omega }}\left( \lambda \right)$ is constant in time, the temporal coefficients ${h_c}$ and $g\left( t \right)$ are unchanged.

### Consequences for spectral fitting and state transition amplitudes

Once the PSI and PSII spectral shapes have been retrieved, any measured fluorescence spectrum can be written as

B.8 $$\matrix{ {} & {{\rm{ChlF}}\left( {t,\lambda } \right) = {F_{{\rm{PSI}}}}\left( t \right)\,{\rm{PSI}}\left( \lambda \right) + {F_{{\rm{PSII}}}}\left( t \right)\,{\rm{PSII}}\left( \lambda \right),} & {} & {} \cr } $$

where ${F_{{\rm{PSI}}}}\left( t \right)$ and ${F_{{\rm{PSII}}}}\left( t \right)$ are the fitted amplitudes of the PSI and PSII fluorescence contributions, respectively.

Using Eqs. (B.5) and (B.6), Eq. (B.8) becomes

B.9 $$\matrix{ {} & {{\rm{ChlF}}\left( {t,\lambda } \right) = {F_{{\rm{PSI}}}}\left( t \right)\,{\rm{\Omega }}\left( \lambda \right)\,{\rm{PS}}{{\rm{I}}_i}\left( \lambda \right) + {F_{{\rm{PSII}}}}\left( t \right)\,{\rm{\Omega }}\left( \lambda \right)\,{\rm{PSI}}{{\rm{I}}_i}\left( \lambda \right)} & {} & {} \cr } $$

and therefore

B.10 $$\matrix{ {} & {{\rm{ChlF}}\left( {t,\lambda } \right) = {\rm{\Omega }}\left( \lambda \right)\left[ {{F_{{\rm{PSI}}}}\left( t \right)\,{\rm{PS}}{{\rm{I}}_i}\left( \lambda \right) + {F_{{\rm{PSII}}}}\left( t \right)\,{\rm{PSI}}{{\rm{I}}_i}\left( \lambda \right)} \right].} & {} & {} \cr } $$

Equation (B.10) shows explicitly that the same optical transfer function ${\rm{\Omega }}\left( \lambda \right)$ multiplies every term of the spectral decomposition. Therefore, the coefficients ${F_{{\rm{PSI}}}}\left( t \right)$ and ${F_{{\rm{PSII}}}}\left( t \right)$ are exactly the same coefficients that describe the decomposition of the corresponding unfiltered spectrum

B.11 $$\matrix{ {} & {{\rm{Chl}}{{\rm{F}}_i}\left( {t,\lambda } \right) = {F_{{\rm{PSI}}}}\left( t \right)\,{\rm{PS}}{{\rm{I}}_i}\left( \lambda \right) + {F_{{\rm{PSII}}}}\left( t \right)\,{\rm{PSI}}{{\rm{I}}_i}\left( \lambda \right).} & {} & {} \cr } $$

The effect of ${\rm{\Omega }}\left( \lambda \right)$ can be factored out from all terms of the fitting equation and does not alter the fitted PSI and PSII amplitudes, provided that the same leaf-specific basis spectra are used and ${\rm{\Omega }}\left( \lambda \right)$ is constant over time. Any fixed scaling convention adopted for the basis spectra does not affect this conclusion, because it does not modify the relative temporal evolution of the fitted amplitudes.

The quantities used to estimate the extent of state transitions are relative changes in the PSI and PSII fluorescence contributions between St1 and St2. For example,

B.12 $$q{T_{{\mathrm{PSII}}}} = \frac{{{F_{{\mathrm{PSII}},{\mathrm{St}}1}} - {F_{{\mathrm{PSII}},{\mathrm{St}}2}}}}{{{F_{{\mathrm{PSII}},{\mathrm{St}}2}}}},$$

and

B.13 $$q{T_{{\mathrm{PSI}}}} = \frac{{{F_{{\mathrm{PSI}},{\mathrm{St}}1}} - {F_{{\mathrm{PSI}},{\mathrm{St}}2}}}}{{{F_{{\mathrm{PSI}},{\mathrm{St}}2}}}}.$$

Since ${\rm{\Omega }}\left( \lambda \right)$ does not change the fitted amplitudes ${F_{{\rm{PSI}}}}\left( t \right)$ and ${F_{{\rm{PSII}}}}\left( t \right)$, it follows directly that it does not change the ratios between the fitted contributions in St1 and St2. Therefore, $q{T_{{\rm{PSI}}}}$ and $q{T_{{\rm{PSII}}}}$ are invariant with respect to fluorescence reabsorption and related radiative-transfer effects, as long as leaf optical properties remain constant during the analysed interval.

### Conditions supporting time-invariant optical weighting

The applicability of the above argument requires experimental conditions under which the internal optical configuration of the leaf is stable over the analysed time window.

Under the present experimental conditions (irradiation by 450–465 nm wavelengths at 30–35 µmol m^−2^ s^−1^), blue light can induce chloroplast movements that are known to affect both leaf absorptance and chlorophyll fluorescence (Brugnoli and Björkman 1992).

However, first, the largest optical transients have been seen during the initial phase after the onset of blue illumination, whereas later observation windows are expected to be less affected once chloroplast distribution has approached a stable configuration (within about 20 min).

Second, far-red light does not induce chloroplast movement (Brugnoli and Björkman 1992; Kagawa and Wada 1996). In addition, fluorescence spectra acquired during the far-red-induced phase were recorded with the far-red source switched off during detector exposure, so that the spectra were always collected under the same blue probe excitation and the same detection geometry. This minimizes acquisition-related changes in excitation penetration during the spectral measurements themselves.

Third, Taylor et al. (2019) showed that absorbance changes associated with blue-light-induced state-transition conditions occur predominantly during the early phase of illumination. This supports the use of later analysis windows, in which both the fluorescence variance structure and the optical configuration of the leaf are expected to be more stable.

Fourth, the red/far-red fluorescence ratio, commonly used as a non-destructive proxy for leaf chlorophyll content, remained unchanged after a complete St1→St2→St1 cycle, indicating that no detectable change in chlorophyll-dependent optical filtering occurred over the duration of the experiment. Although this ratio is not a full radiative-transfer measurement, its stability is consistent with the assumption that the effective leaf optical conditions relevant to fluorescence detection remained unchanged on the timescale of the state-transition analysis.

### Experimental verification using leaves with contrasting chlorophyll content

As an independent test of the above interpretation, the PCA-based retrieval was applied to two groups of tobacco leaves with markedly different chlorophyll contents.

Leaves were selected based on their F_735_/F_700_ ratio—widely used as a non‑destructive proxy for leaf chlorophyll content (Buschmann 2007)—and converted to chlorophyll concentration following the relationship reported by Gitelson et al. (1999). Table 3 reports the average F_735_/F_700_ values and the corresponding chlorophyll contents (Chl = 634·F_735_/F_700_ − 391). The two groups differed substantially, with mean Chl contents of approximately 55 µg cm^−^^2^ (Higher‑Chl leaves) and 28 µg cm^−^^2^ (Lower‑Chl leaves).

**Table 3:** Leaf far-red/red fluorescence ratios and derived Chl content by the relation: Chl = 634·F_73__5_/F_700_–391, (Gitelson et al. 1999)). Extent of state transition (qT) determined by the change in the two photosystem ChlF components, as (ChlF_1_-ChlF_2_)/ChlF_2_, during the St2→St1 transition. Mean ± SD, *n* = 3.*

	F_735_/F_700_	Chl (µg cm^−2^)	qT (%)	
			PSI	PSII
Higher Chl	1.48 ± 0.02 a	54.7 ± 1.3 a	20.0 ± 2.6	20.1 ± 1.2
Lower Chl	1.06 ± 0.09 b	28.1 ± 5.8 b	19.8 ± 2.8	17.7 ± 1.5

*For F_735_/F_700_ and Chl, significant differences in values between the two leaf groups are marked by different lower-case letters. For qT, no statistically significant difference between the two photosystem components was observed. Statistical comparisons were performed by the Welch’s t-test

As expected, the retrieved PSI and PSII fluorescence spectral shapes differed between the two groups: reduced chlorophyll content decreased the extent of fluorescence reabsorption, leading to an enhancement of the 685‑nm red band relative to the 740‑nm far‑red band in both photosystems (Fig. 11).

Importantly, however, the magnitude of the relative fluorescence change between St1 and St2 (Table 3) was not significantly affected by chlorophyll content. For PSI, the St1→St2 variation was 20.0% ± 2.6% and 19.8% ± 2.8% (*p* = 0.925) in higher‑ and lower‑Chl leaves, respectively; for PSII, the corresponding values were 20.1% ± 1.2% and 17.7% ± 1.5% (*p* = 0.10). The PSI and PSII fluorescence kinetics recorded during ST were likewise very similar for the two chlorophyll levels (Fig. 12).

These results indicate that, although chlorophyll reabsorption modifies the absolute spectral shapes retrieved by PCA, it affects all emission spectra in a comparable, wavelength‑dependent manner. Consequently, it does not introduce differential distortions among the spectra and therefore does not bias the PCA‑based retrieval of PSI and PSII contributions during state transitions.

## Appendix C. Quantitative relationship between photosystem ChlF and light–absorption cross–section changes (including PSII→PSI spillover)

This Appendix reports the formal derivation underlying the qualitative statements given in the main text, based on early excitation energy distribution models and on the spillover decomposition proposed by Terashima et al. (2025).

### Definitions and assumptions

We adopt the scheme in Fig. 7.

Symbols (state dependent where specified):

•$${F_{PSI}}$$ = PSI fluorescence

•$${F_{PSII}}$$ = PSII fluorescence

•${A^I}$ = absorption cross section of the PSI intrinsic antenna.

•${A^{II}}$ = absorption cross section of the PSII antenna (LHCII).•

$${A^{II}} = A_a^{II} + A_b^{II}$$

where:

$A_a^{II}$ = “stable” PSII antenna contribution, containing the strongly bound LHCII-S + moderately bound LHCII-M.

$A_b^{II}$ = “mobile” antenna contribution, loosely bound LHCII-L, that migrates betweenPSII and PSI during ST.

•${\phi _{II \to I}}$ = quantum yield of PSII→PSI spillover (excitation transfer).

•${\phi _{{F_{II}}}}$ = PSII fluorescence quantum yield.

•${\phi _{{F_I}}}$ = PSI fluorescence quantum yield.

For each photosystem, we assume that fluorescence is proportional to: (absorbed excitation), reduced or increased by spillover in PSII and PSI, respectively, × (fluorescence quantum yield), and the state dependence is captured by whether $A_b^{II}$ is associated with PSII (St1) or PSI (St2).

### PSII fluorescence in St1 and St2

In St1, PSII antenna includes both stable and mobile components, thus:

C.1 $$F_{PSII}^{St1} \propto {A^{II}}\left( {1 - {\phi _{II \to I}}} \right){\phi _{{F_{II}}}}$$

that is

C.2 $$F_{PSII}^{St1} \propto [\left( {A_a^{II} + A_b^{II}} \right)\left( {1 - {\phi _{II \to I}}} \right)]{\phi _{{F_{II}}}}$$

In St2, the mobile fraction is detached from PSII; PSII retains only $A_a^{II}$:

C.3 $$F_{PSII}^{St2} \propto A_a^{II}\left( {1 - {\phi _{II \to I}}} \right){\phi _{{F_{II}}}}$$

Subtracting (C.3) from (C.2), we derive

C.4 $$\Delta F_{PSII}^{St1 - St2} = F_{PSII}^{St1} - F_{PSII}^{St2} \propto A_b^{II}\left( {1 - {\phi _{II \to I}}} \right){\phi _{{F_{II}}}}$$

And dividing (C.4) by (C.3) yields:

C.5 $${{\Delta F_{PSII}^{St1 - St2}} \over {F_{PSII}^{St2}}} = {{A_b^{II}} \over {A_a^{II}}}$$

**Interpretation:**the relative change of the retrieved PSII fluorescence component between St1 and St2 provides ${{A_b^{II}} \over {A_a^{II}}},$ i.e. the mobile antenna fraction relative to the stable PSII antenna.

### PSI fluorescence in St1 and St2 (explicit spillover term)

PSI fluorescence includes: (i) an intrinsic term due to excitation delivered from its own antenna, (ii) an additional term due to spillover from PSII.

In St1:

C.6 $$F_{PSI}^{St1} \propto \left( {{A^I} + {A^{II}}{\phi _{II \to I}}} \right){\phi _{{F_I}}}$$

In St2, $A_b^{II}$ is attached to PSI, while PSII retains $A_a^{II}$; PSI can still receive spillover from PSII proportional to $A_a^{II}$:

C.7 $$F_{PSI}^{St2} \propto ({A^I} + A_b^{II} + A_a^{II}{\phi _{II \to I}}){\phi _{{F_I}}}$$

Subtracting (C.6) from (C.7) gives:

C.8 $$\Delta F_{PSI}^{St2 - St1} = F_{PSI}^{St2} - F_{PSI}^{St1} \propto A_b^{II}\left( {1 - {\phi _{II \to I}}} \right){\phi _{{F_I}}}$$

Dividing by $F_{PSI}^{St2}$ provides:

C.9 $${{\Delta F_{PSI}^{St2 - St1}} \over {F_{PSI}^{St2}}} = {{A_b^{II}\left( {1 - {\phi _{II \to I}}} \right)} \over {{A^I} + A_b^{II} + A_a^{II}{\phi _{II \to I}}}}$$

Eqs. (C.9) contains multiple unknowns (${A^I}$, ${\phi _{II \to I}}$, and LHCII partitioning terms), and therefore cannot be uniquely solved from fluorescence changes alone.

### PSI fluorescence α/β decomposition (Terashima formalism) and spillover correction factor

Following Terashima et al. (2025), PSI fluorescence is written as the sum of:

•${F_{PSI, \alpha }}$ = intrinsic PSI fluorescence (excitation delivered to PSI from its antenna).

•${F_{PSI,\beta }}$ = PSI fluorescence due to spillover from PSII.

Thus:

C.10 $$F_{PSI}^{St1} = F_{PSI,\alpha }^{St1} + F_{PSI,\beta }^{St1} = F_{PSI,\alpha }^{St1}\left( {1 + {{F_{PSI,\beta }^{St1}} \over {F_{PSI,\alpha }^{St1}}}} \right)$$

C.11 $$F_{PSI}^{St2} = F_{PSI,\alpha }^{St2} + F_{PSI,\beta }^{St2} = F_{PSI,\alpha }^{St2}\left( {1 + {{F_{PSI,\beta }^{St2}} \over {F_{PSI,\alpha }^{St2}}}} \right)$$

and

C.12 $${{F_{PSI}^{St2}} \over {F_{PSI}^{St1}}} = {{F_{PSI,\alpha }^{St2}} \over {F_{PSI,\alpha }^{St1}}} \cdot {{\left( {1 + R_{\beta /\alpha }^{St2}} \right)} \over {\left( {1 + R_{\beta /\alpha }^{St1}} \right)}} = {{F_{PSI,\alpha }^{St2}} \over {F_{PSI,\alpha }^{St1}}} \cdot {C_{spillover}}$$

where $R_{\beta /\alpha }^{St2}$and $R_{\beta /\alpha }^{St1}$ refer to the ratio between the β and α F_PSI_ contributions at St2 and St1, respectively.

**Interpretation:**the measured ratio ${{F_{PSI}^{St2}} \over {F_{PSI}^{St1}}}$ equals the intrinsic ratio ${{F_{PSI,\alpha }^{St2}} \over {F_{PSI,\alpha }^{St1}}}$ (proportional to the PSI absorption cross section ratio) multiplied by a spillover dependent correction factor: ${C_{spillover}} = {{\left( {1 + R_{\beta /\alpha }^{St2}} \right)} \over {\left( {1 + R_{\beta /\alpha }^{St1}} \right)}}$.

Terashima et al. (2025) provided estimates of spillover contributions (${F_{PSI,\beta }}$) relative to the total PSI (${F_{PSI,\beta }} + {F_{PSI,\alpha }}$) signal at St1 and St2 across species and growth conditions. From these data, by few simple algebraic steps it is possible to derive ${F_{{\rm{PSI}},\beta }}/{F_{{\rm{PSI}},\alpha }}$ and then the $\left( {1 + {{F_{PSI,\beta }^{St2}} \over {F_{PSI,\alpha }^{St2}}}} \right)/\left( {1 + {{F_{PSI,\beta }^{St1}} \over {F_{PSI,\alpha }^{St1}}}} \right) =$ ${{\left( {1 + R_{\beta /\alpha }^{St2}} \right)} \over {\left( {1 + R_{\beta /\alpha }^{St1}} \right)}} =$ C_spillover_

These values represent the maximum spillover contribution to PSI fluorescence, as they were obtained under conditions of closed reaction centres and low NPQ. Conversely, in the present study, reaction centres at the steady state of both St1 and St2 are predominantly in the oxidized form (Table 1), which reduces the spillover contribution to ${F_{PSI}}$ compared with the conditions used by Terashima et al. (2025). NPQ was almost insignificant (between 0.22–0.31 for the A and H lines at St1 and St2, Table 1), and its effect on ${\phi _{II \to I}}$ can therefore be neglected. Considering the Terashima’s data, and the fact that the yields of PSII to PSI energy transfer determined by Strasser and Butler (1977a, c) at F_0_ were 2–2.5 times lower than those at F_m_, we rescaled ${F_{{\rm{PSI}},\beta }}$ to make it adequate to the condition of open reaction centres and derived C_spillover_ to fall within the range 0.92–0.97.

Therefore, the intrinsic (antenna driven) ratio can be estimated as:

C.13 $${{F_{PSI,\alpha }^{St2}} \over {F_{PSI,\alpha }^{St1}}} = \cdot {{F_{PSI}^{St2}} \over {F_{PSI}^{St1}}} \cdot {1 \over {{C_{spillover}}}} \approx {{F_{PSI}^{St2}} \over {F_{PSI}^{St1}}} \cdot {1 \over {\left( {0.92\,to\,0.97} \right)}}$$

Applying this correction yields an estimated PSI absorption cross section ratio between St2 and St1 of approximately 1.27 to 1.34, i.e. a 27–34% increase in the cross section, reported in the main text as ~31% for conciseness.
