# Supplementary material for: Improved state‑transition analysis in tobacco lines differing in isoprene emission via spectral‑retrieval separation of PSI and PSII fluorescence at physiological temperature
Source: Photosynth Res. 2026 Jul 23;164(4):41. doi: 10.1007/s11120-026-01227-z (PMC13396053; doi:10.1007/s11120-026-01227-z)
Supplement: Supplementary file 1 — Supplementary Material 1 [file 11120_2026_1227_MOESM1_ESM.docx]

**Supplementary Information**

**Improved state‑transition analysis in tobacco lines differing in isoprene emission via spectral‑retrieval separation of PSI and PSII fluorescence at physiological temperature**

Lorenzo Palombi^1^[0000-0001-9219-564X](https://orcid.org/0000-0001-9219-564X), Susanna Pollastri^2^[0000-0001-5136-1488](https://orcid.org/0000-0001-5136-1488), Francesco Loreto^2,^[^3^0000-0002-9171-2681](https://orcid.org/0000-0002-9171-2681), Giovanni Agati^1^[0000-0003-0855-9389](https://orcid.org/0000-0003-0855-9389)

^1^Institute of Applied Physics “Nello Carrara” (IFAC), National Research Council (CNR), Sesto Fiorentino, 50019 Florence, Italy

^2^Institute for Sustainable Plant Protection (IPSP), National Research Council (CNR), Sesto Fiorentino, 50019 Florence, Italy

^3^Department of Biology, University of Naples Federico II, 80126 Naples, Italy


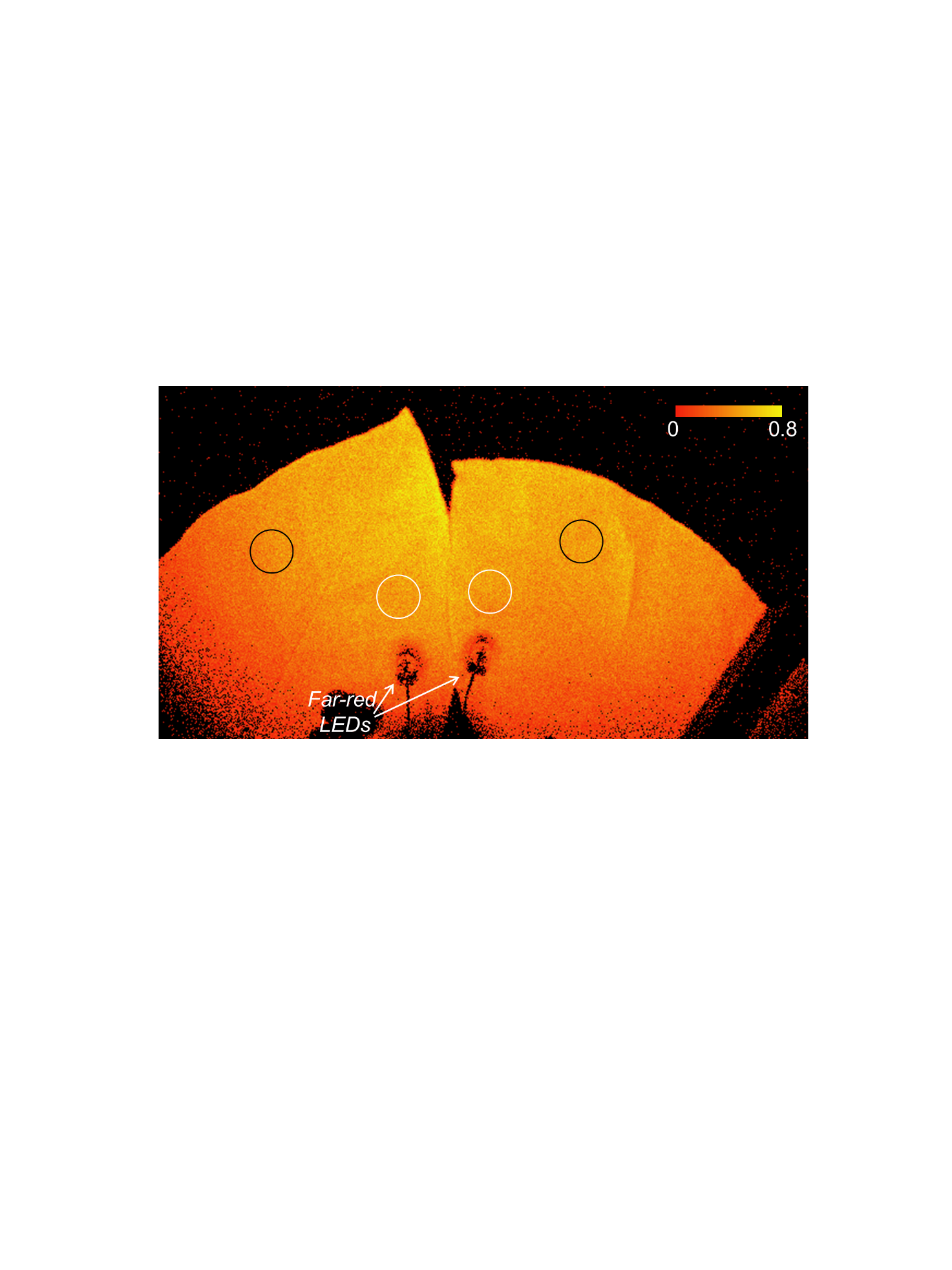


**Figure S1** Representative chlorophyll (Chl) fluorescence images of two tobacco leaves acquired by the MAXI-Imaging Pulse-Amplitude Modulation (PAM) during state transition. The left and right leaves belonged to the isoprene-emitting (H) and non-emitting (A) tobacco plants, respectively. The samples were homogeneously illuminated by 450 nm light emitting diodes (LEDs). Far-red LEDs at 720 nm were additionally employed to induce St2 to St1 transition in the area-of-interest (AOI) indicated by the white circles. The black circles indicate the AOI used to acquire the Chl fluorescence under constant photosystem II (PSII) light, as controls.


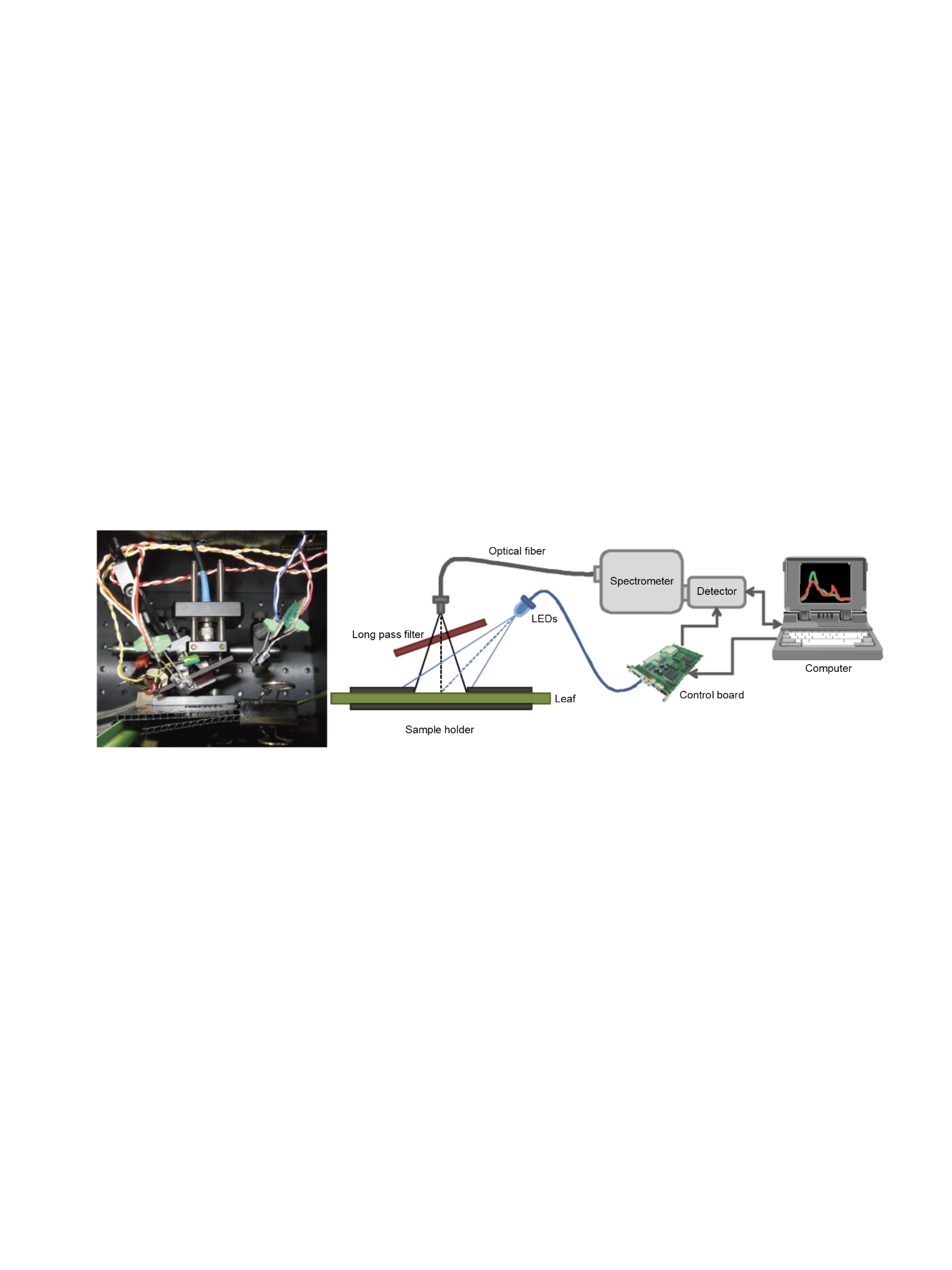


**Figure S2** On the left, top view of the fluorescence excitation and acquisition zone of the experimental apparatus, as schematized on the right.


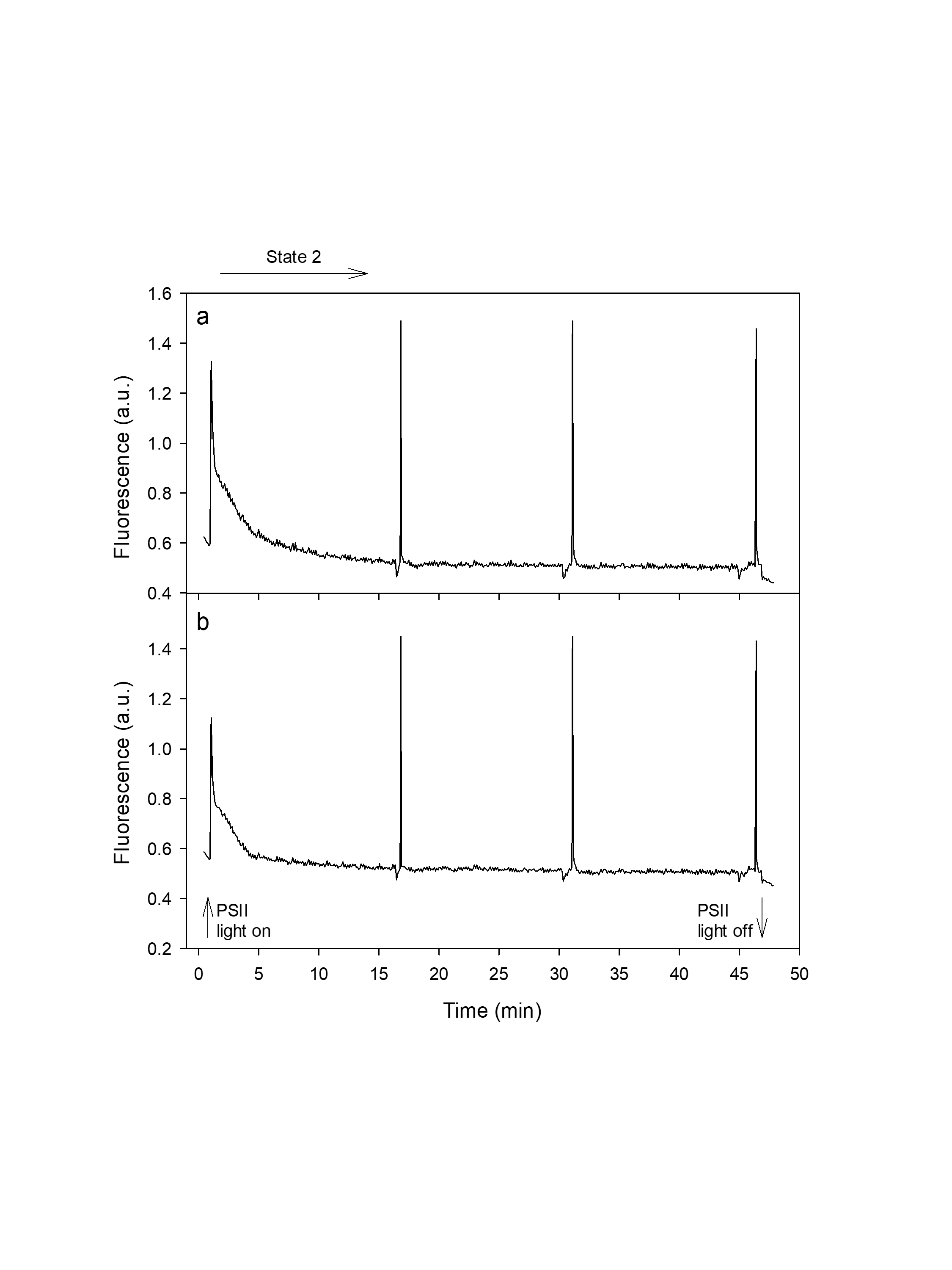


**Figure S3** Representative kinetics of the chlorophyll fluorescence (ChlF) measured by the Imaging-PAM on the area of interest (AOI) of control leaves, unirradiated by the photosystem I (PSI) light. Once ChlF reached the steady state of the first Kautsky kinetics it did not change further remaining the photosystems in State 2 (St2) during state transition in a transgenic line azygous for isoprene synthase (A) and a transgenic line homozygous for isoprene synthase (H) of *Nicotiana tabacum* (cv. Samsun).


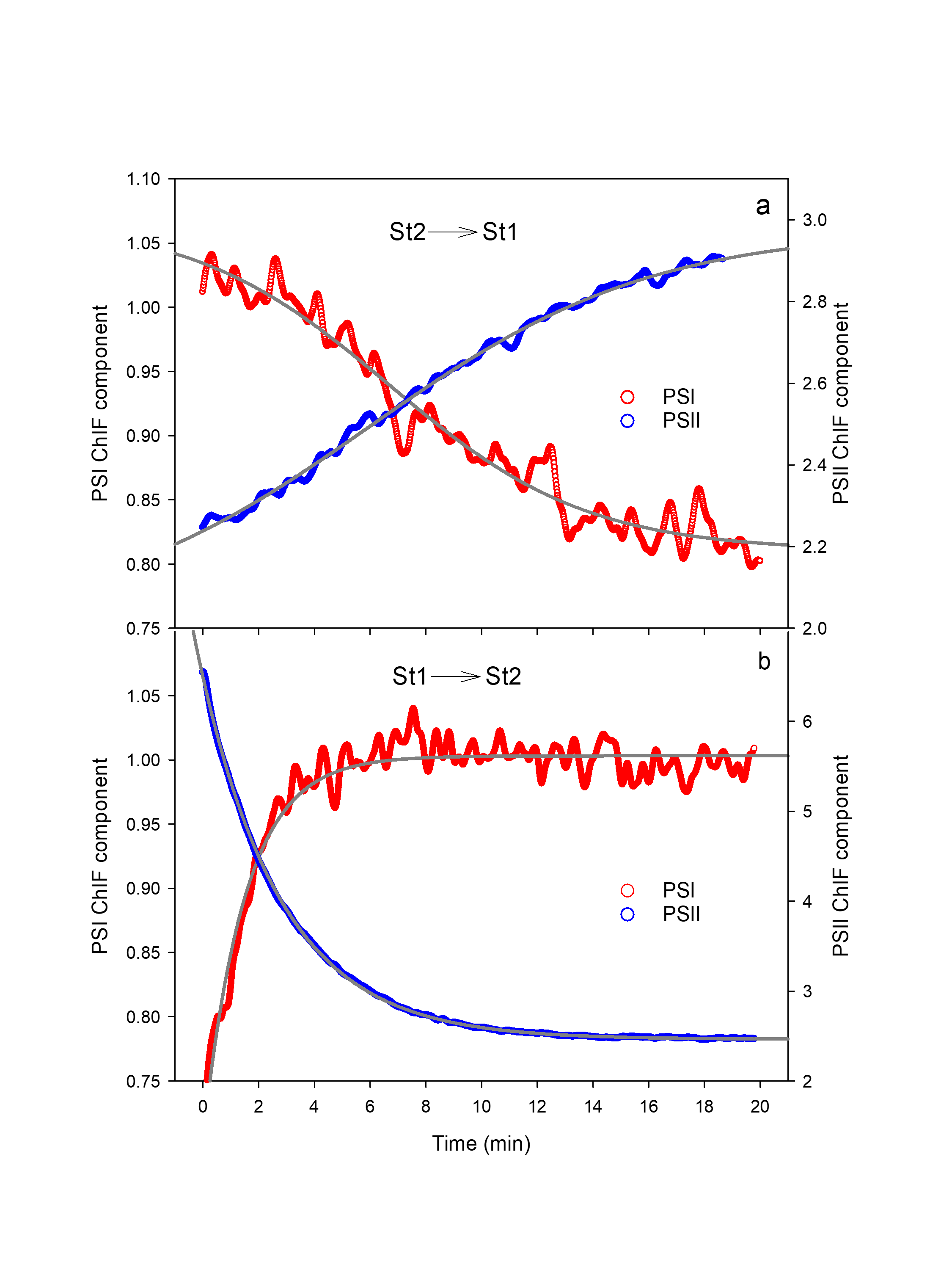


**Figure S4** Curve fitting of the PSI and PSII ChlF components kinetics during St2→St1 (A) and St1→St2 (B) transitions of a *N. tabacum* A line leaf. In A, sigmoid curves with equation f = y0+a/(1+exp(-(x-x0)/b)) was used. R^2^ of fitting was 0.964 and 0.997 for PSI and PSII respectively. In B, a rising exponential curve with equation f = y0+a*(1-exp(-b*x)) was used for PSI (R^2^ = 0.948) and a decreasing exponential curve with equation f = y0+a*exp(-b*x) was used for PSII (R^2^ = 0.999).


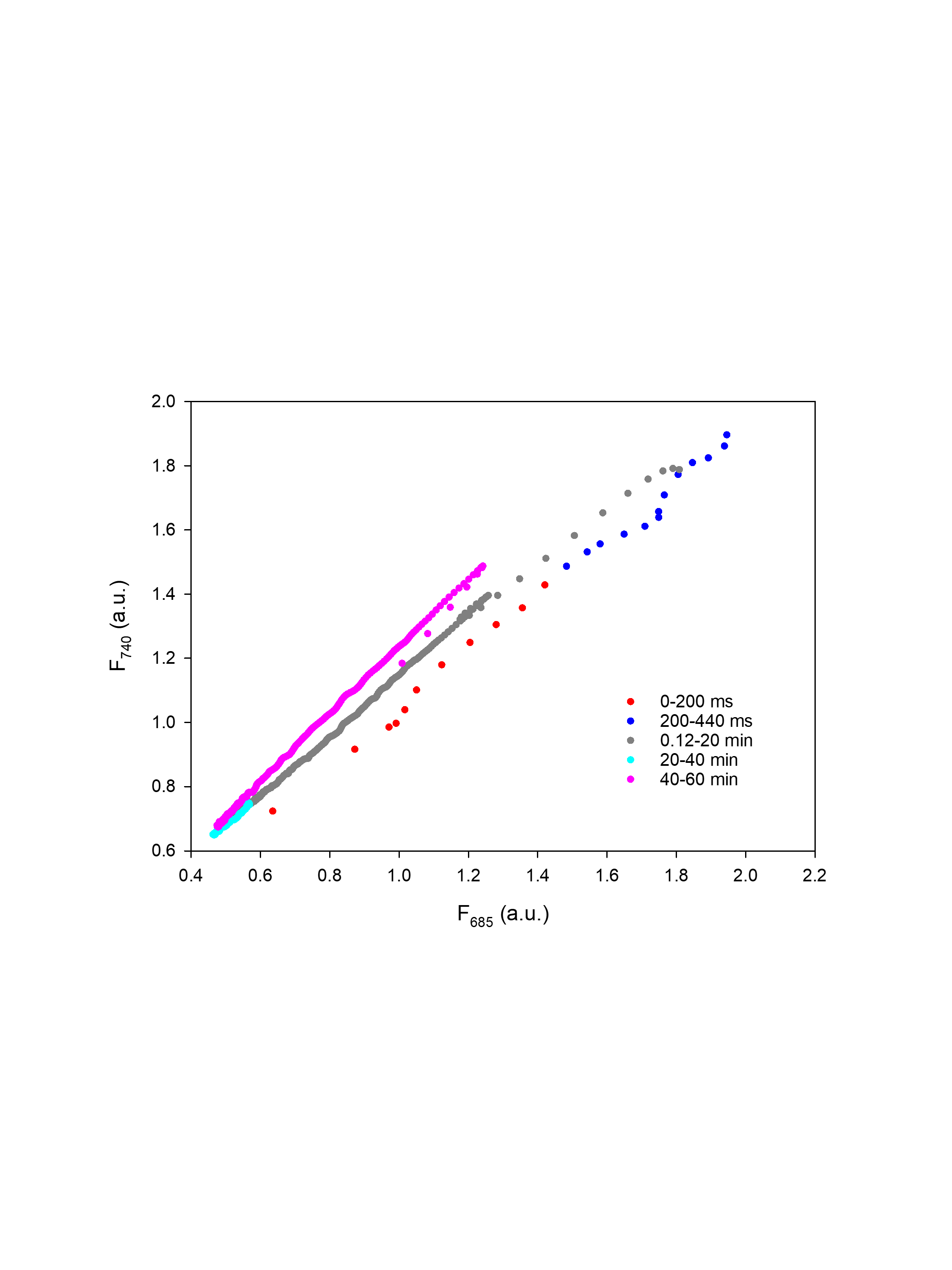


**Fig.S5** F_740_ vs F_685_ during different phases of the Chl fluorescence induction curves in a tobacco H line leaf: the initial fast rising of the Kautsky kinetics origin-inflection (OI) point photochemical phase (red dots, 0-200 ms); a second rising phase (blue dots, 200-440 ms) and the decrease phase of the Kautsky kinetics reaching a St2 steady level (grey dots, 0.12-20 min); the St2 to St1 transition initial (cyan dots, 20-40 min); the post-IR phase from St1 to St2 (magenta dots, 40-60 min). The plot of the last temporal phase (60-80 min) was overlapped to the first St2 to St1 transition (cyan dots) and it is not reported for clarity. Data refer to the acquisitions on a tobacco H line leaf.
